# Rapid in-EPON CLEM: Combining fast and efficient labeling of self-labeling enzyme tags with EM-resistant Janelia Fluor dyes and StayGold

**DOI:** 10.1016/j.heliyon.2024.e28055

**Published:** 2024-03-18

**Authors:** Rico Franzkoch, Sabrina Wilkening, Viktoria Liss, Michael Holtmannspötter, Rainer Kurre, Olympia E. Psathaki, Michael Hensel

**Affiliations:** a Abt. Mikrobiologie, Universität Osnabrück, Osnabrück, Germany; b IBiOs – Integrated Bioimaging Facility Osnabrück, Germany; c CellNanOs – Center for Cellular Nanoanalytics Osnabrück, Germany

**Keywords:** In-resin correlative light and electron microscopy, Post-embedding CLEM, Self-labeling enzyme tags, Janelia fluor dyes, HaloTag

## Abstract

Correlative light and electron microscopy (CLEM) combines light microscopy (LM) of fluorescent samples to ultrastructural analyses by electron microscopy (EM). Pre-embedding CLEM often suffers from inaccurate correlation between LM and EM modalities. Post-embedding CLEM enables precise registration of structures directly on EM sections, but requires fluorescent markers withstanding EM sample preparation, especially osmium tetroxide fixation, dehydration and EPON embedding. Most fluorescent proteins (FPs) lose their fluorescence during such conventional embedding (CE), but synthetic dyes represent promising alternatives as their stability exceeds those of FP. We analyzed various Janelia Fluor dyes and TMR conjugated to ligands for self-labeling enzymes, such as HaloTag, for fluorescence preservation after CE. We show that TMR, JF525, JF549, JFX549 and JFX554 retain fluorescence, with JFX549 and JFX554 yielding best results overall, also allowing integration of high-pressure freezing and freeze substitution. Furthermore, we found the recently published FP StayGold to resist CE, facilitating dual-fluorescence in-resin CLEM.

## 1 Introduction

Transmission electron microscopy (TEM) with its near atomic spatial resolution has been used successfully for decades to unravel the ultrastructural architecture of different cellular components [1]. The chemicals used in sample preparation for EM interact with and thereby stain a variety of cellular content enabling the visualization of nearly all cellular components at the same time [2]. This results in a unique reference space. The localization of specific proteins on TEM sections is possible for instance via immunogold labeling, but needs specifically adapted protocols, suitable antibodies, a very careful interpretation of the results, and is limited through the penetration depth of the antibodies [[3], [4], [5]]. In light microscopy (LM) cellular structures or proteins can be directly labeled by fluorescent proteins (FP) or dyes with high efficiency and the dynamics inside living cells can be easily monitored [6]. Due to the comparably low resolution of LM and the lack of ultrastructural context, precise and unambiguous identification of the structures underlying the fluorescence signal remains challenging. The combination of both imaging modalities as correlative light and electron microscopy (CLEM) is able to overcome the individual limitations and presents a powerful tool gaining more and more recognition in cellular biology during the recent years [[7], [8], [9]]. Depending on the biological question to be addressed, several CLEM approaches were developed. These can be distinguished in workflows conducting the LM prior to embedding (pre-embedding), after the embedding step (post-embedding), or employing a combination of both. During pre-embedding CLEM, the sample is either imaged in living or aldehyde-fixed state. The former allows for visualization of cellular dynamics and does not impede the intensity of fluorescence signals. Subsequent to LM, EM sample preparation is performed consisting of post-fixation with osmium tetroxide, dehydration, and final embedding in resin [9,10]. Such steps are known to induce artifacts including shrinkage and extraction of cellular material [11]. Such artifacts can severely compromise correlation of LM and EM images. And even without preparation artifacts, correlation for pre-embedding CLEM is very challenging, especially since Z resolution in LM is ca. 500 nm, thus about 10-times lower than for ultrathin resin sections of typical thickness of 50–70 nm. To overcome these limitations, post-embedding LM in combination with high-pressure freezing (HPF) and freeze substitution (FS) is an emerging alternative. For this, LM and EM modalities are registered on the same section, resulting in very high accuracy of correlation [12,13]. Furthermore, ultrastructural preservation is highly improved due to HPF and FS [11]. Drawbacks of such techniques are the need for sophisticated, expensive equipment, and use of methacrylate resins (e. g. Lowicryl HM20) which also limit contrast and have inferior properties in sectioning compared to epoxy resins. Also, the increased complexity of workflows demands highly experienced personnel [14,15]. Certain staining and fixation agents like osmium tetroxide have to be completely omitted, or can only be used in low concentrations such as 0.1% uranyl acetate, since these chemicals have a strong impact on the fluorescence [12,16,17].

Post-embedding CLEM was also achieved with immunolabeled Tokuyasu sections [18,19], or thawed cryosections of cells expressing fluorescent proteins or pulse/chased with fluid tracers [20]. However, such approaches also omit osmium tetroxide and in particular dehydration, resulting in potentially excellent membrane preservation, but provide only low levels of contrast in EM modalities. Furthermore, cryo-sectioning is technically more challenging than performing ultramicrotomy at room temperature, and may induce sectioning artifacts. Even though fluorescence is less impaired by these preparation methods, the more complicated sectioning procedure in combination with less membrane contrast can make correlation more difficult and requires more experience than preparation of EM samples by conventional embedding (CE). A recent approach to simplify post-embedding CLEM is the use of fluorescent proteins which can, to some degree, tolerate the quenching properties of CE sample preparation including osmium tetroxide fixation and dehydration at room temperature. For this, special variants of the Eos-FP like mEosEM were engineered or standard fluorescent proteins like mWasabi, mKate2 or mScarlet-H were tested for their resistance to TEM sample preparation [14,16,21,22]. However, many of these proteins seem to retain only comparably weak fluorescence signals and low signal to background ratio after EPON embedding, and require protocols with reduced osmium tetroxide concentration [22].

An alternative to fluorescent proteins are fluorophores such as tetramethylrhodamine (TMR) that can be coupled to ligands that are specifically, covalently, and irreversibly bound by self-labeling enzyme tags (SLE) such as HaloTag, SNAP-tag or CLIP-tag [6]. This system allows for rapid and precise labeling of tagged cellular structures, and is compatible with super-resolution microscopy (SRM). Regarding the use of CE protocols with EPON embedding, systematic analysis of performance of available fluorophore-conjugated SLE ligands is missing. Only few publications employed dyes in such workflows, suggesting that they might be promising alternatives to fluorescent proteins [23,24]. Müller et al. showed that fluorescent signals of insulin granules labeled with TMR-Star and 505-Star are visible after conventional EPON embedding, and also after HPF and FS. However, in this CE protocol only low concentration (0.1%) of osmium tetroxide in combination with uranyl acetate was used for 30 min, which might not be sufficient to preserve all ultrastructural details. Additionally, very high concentrations (6 μM) of dyes and long incubation times (overnight) were employed, making this a more costly and time-consuming addition to the protocol. Sanada et al. and Tanida et al. employed higher osmium concentrations, but used cell-impermeable dyes such as DyLight 549 or HiLyte 555 which require cell permeabilization, and thereby possibly diminishes ultrastructural quality.

As we see great potential in a rapid in-resin CLEM approach that combines fast and efficient labeling of SLE tags with EM-resistant dyes, and due to the limited or partly contradictory published data, we set out to systematically evaluate the performance of various fluorescent SLE ligand conjugated dyes after CE sample preparation with osmium tetroxide fixation, dehydration and EPON embedding. We aimed for short labeling times to minimize additional workload in sample preparation, and use of low concentrations of dyes. For this, we especially focused on Janelia Fluor dyes since these were specifically engineered to exhibit the highest brightness and photostability outperforming other current dyes [25].

## 2 Results

### 2.1 Rapid and simple conventional sample preparation for in-resin CLEM

In this study, we evaluated the fluorescence retainment of TMR and various Janelia Fluor dyes conjugated to the HaloTag ligand (HTL) after labeling intracellular proteins of interest tagged with the HaloTag and conventional EM sample preparation with EPON embedding for TEM. Janelia Fluor dyes were specifically engineered to exhibit the highest brightness and photostability outperforming other current dyes [[25], [26], [27]]. For comparison we chose HTL-TMR as standard available in our lab. The dyes tested, namely the green fluorescent JF479, the red fluorescent JF549, JFX549, JFX554 and JF585, and the far-red fluorescent JF646, JFX646 and JFX650, are listed in Table 4 with their properties. In Fig. S1, the chemical structures and the origin of rhodamine-derived Janelia Fluor dyes can be found (from now on the term dye will be used to describe the combination of a dye with its HTL-ligand if not stated otherwise).

**Table 1:** Dyes used for staining.

Dye	Supplier
JF479, JF525, JF549, JFX549, JFX554, JF585, JF646, JFX646, JFX650 conjugated to HaloTag ligand	L. D. Lavis, Janelia Research Campus
JF525 conjugated to BG SNAP-tag ligand	L. D. Lavis, Janelia Research Campus
HaloTag® TMR Ligand	Promega
Dextran-Alexa Fluor® 647	Life Technologies

**Table 2:** Cell lines used in this study.

Designation	relevant characteristics	source/reference
HeLa Tom20-HaloTag	HeLa WT cells, stably transfected, Tom20-HaloTag expression	Piehler group, Biophysics UOS [57],
HeLa LAMP1-HaloTag	HeLa WT cells, stably transfected, LAMP1-HaloTag expression	this study
HeLa LAMP1-GFP	HeLa WT cells, stably transfected, with LAMP1-GFP expression	Hensel group [9],
HEK293T/17	human embryonic kidney epithelial cells	Hensel group [38],

**Table 3:** Plasmids used in this study.

Designation	Purpose	genotype	source/reference
pFPV25.1	bacterial expression	P*rpsM*::eGFP mut3	AG Hensel, Microbiology, UOS
p4564	mammalian expression	*sseG*::HaloTag	AG Hensel, Microbiology, UOS
p6032	mammalian expression	Golgi:HaloTag	AG Hensel, Microbiology, UOS
pCx36-SNAP	mammalian expression	Cx36:SNAP-tag	[38]
pcDNA3/er-(n2)oxStayGold(c4)	mammalian expression	KDEL::StayGold	[40], Addgene 185,822

**Table 4:** Physical properties of the fluorophores analyzed in this study.

Fluorophore	*λ*_max_	λem	ε (M − 1 cm-1 × 103)	Φ	Properties
JF479	479 nm	517 nm	47.9	0.62	lower cell permeability
JF525	525 nm	549 nm	94.0	0.91	extraordinary cell and tissue permeability
TMR	555 nm	585 nm	89.0	0.41	sensitive to photobleaching
JF549	549 nm	571 nm	101.0	0.88	direct analog of TMR
JFX549	548 nm	570 nm	96.7	0.86	modestly higher brightness and photostability than JF549
JFX554	554 nm	576 nm	104.0	0.8	brightest and most photostable red dye
JF585	585 nm	609 nm	1.5	0.78	fluorogenic dye (80x increase), slightly lower cell permeability
JF646	646 nm	664 nm	5.6	0.54	modestly fluorogenic dye
JFX646	645 nm	662 nm	8.6	0.54	modestly higher brightness and photostability than JF646
JFX650	650 nm	667 nm	17.6	0.53	brightest and most photostable far-red dye

Λ_max_: maximum absorption, λ_em_: fluorescence emission maximum, ε: extinction coefficient, Φ: quantum yield. ε indicates the photostability of a dye, while Φ describes the brightness of the respective dye. All information taken from Grimm et al. [26], Presman et al. [58], Kompa et al. [59], and https://janeliamaterials.azurewebsites.net.

In an initial examination of the dyes, we checked the intracellular fluorescence only. The epithelial cell line HeLa stably transfected for Tom20-HaloTag expression was used for labeling experiments throughout this paper. The optimal concentration and labeling duration for each dye depend on many different conditions, like cell type, protein tagged, SLE used, ligand and dye itself. Previous studies used comparable dyes for LM applications at concentrations between 10 and 500 nM with staining for 10–30 min [25,[28], [29], [30], [31], [32]], or concentrations of dyes as high as 1 μM [33,34], to label candidate protein-HaloTag conjugates. Apart from certain experiments, we used all dyes at concentration of 100 nM with staining for 30 min as standard to achieve comparable conditions. We previously reported these labeling conditions for HTL-TMR to provide sufficient fluorescence signals in LM [6], and HTL-TMR here serves as standard for identification of additional suitable dyes. HeLa Tom20-HaloTag cells were stained with 100 nM of each dye for 30 min before fixation with 3% PFA for 15 min. Subsequent imaging by confocal laser-scanning microscopy (CLSM) mostly showed fluorescence signals matching the distribution of mitochondria, and quantification of fluorescence revealed signals being clearly distinct from the background (Fig. S3). Only JF479 and JF585 yield low fluorescence signals that impaired identification of labeled mitochondria. The fluorogenic fluorophore JF585 showed signal-to-background ratio (S/B) comparable to the other red dyes. Also noteworthy is the increased fluorescence signal of JFX646 compared to JF646, fluorophores which only differ in the deuteration of JFX646.

**Fig. 1:**
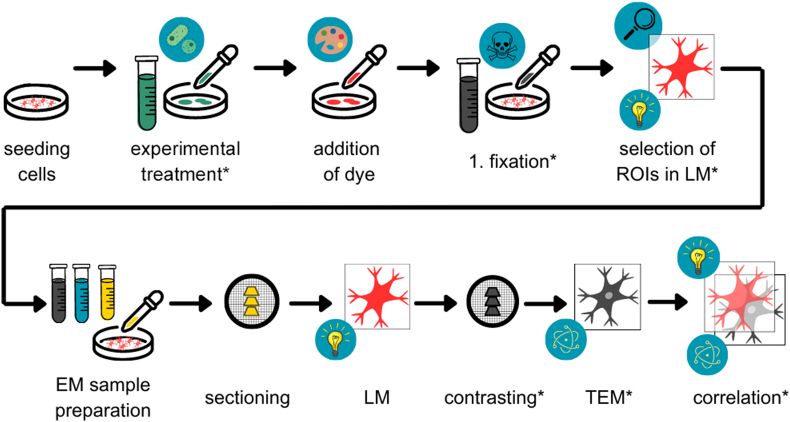
Workflow of sample preparation for in-resin CLEM. HeLa cells expressing self-labeling enzyme (SLE) fusions were stained with ligand-conjugated dyes before first fixation with glutaraldehyde (GA) and paraformaldehyde (PFA). If indicated, cells were experimentally treated, e. g. infected, transfected, or pulse/chased with fluid tracers, Cells of interest were selected by light microscopy (LM). Subsequently, samples were prepared for electron microscopy (EM) by further fixation with GA, post-fixation with osmium tetroxide, dehydration in ethanol, and embedding in EPON. Sections were generated and registered by widefield LM, followed by contrasting and imaging by transmission electron microscopy (TEM). Finally, the LM and EM modalities were overlaid and correlated. *Steps marked by asterisks were omitted for quantitative comparison of various dyes.

As we aimed for a CLEM approach that can easily be used in any laboratory without additional equipment, a conventional EM embedding protocol with slight adaptations was used (Fig. 1). After labeling HeLa Tom20-HaloTag cells with various dyes in the respective concentration, cells were either first fixed with 0.2% GA and 3% PFA for locating ROIs in LM if necessary or directly subjected to EM preparation. Subsequent EM sample preparation was comprised of fixation with 2.5% GA for 1 h, post-fixation with osmium tetroxide and potassium hexacyanoferrate, dehydration in a graded ethanol series, incubation in anhydrous acetone, infiltration with and polymerization of EPON. To minimize quenching of the fluorescence by osmium tetroxide, its incubation time was reduced to 30 min during post-fixation, instead of 1 h as de scribed in standard embedding protocols [9,10]. We checked the membrane preservation and contrast after this sample preparation protocol and found well preserved ultrastructure (Fig. S2). Different cell organelles such as mitochondria, nucleus, lysosomes, Golgi and also membrane contact sites are well visible even after a shortened osmium incubation (Fig. S2). Finally, sections of 250 nm or 100 nm were cut and viewed in widefield LM followed by contrasting with 3% uranyl acetate for 30 min and 2% lead citrate for 20 min. The images acquired by TEM were finally overlaid and correlated with the fluorescence images of the sections.

### 2.2 Several Janelia Fluor dyes retain fluorescence within thin EPON sections after conventional sample preparation

**Fig. 2:**
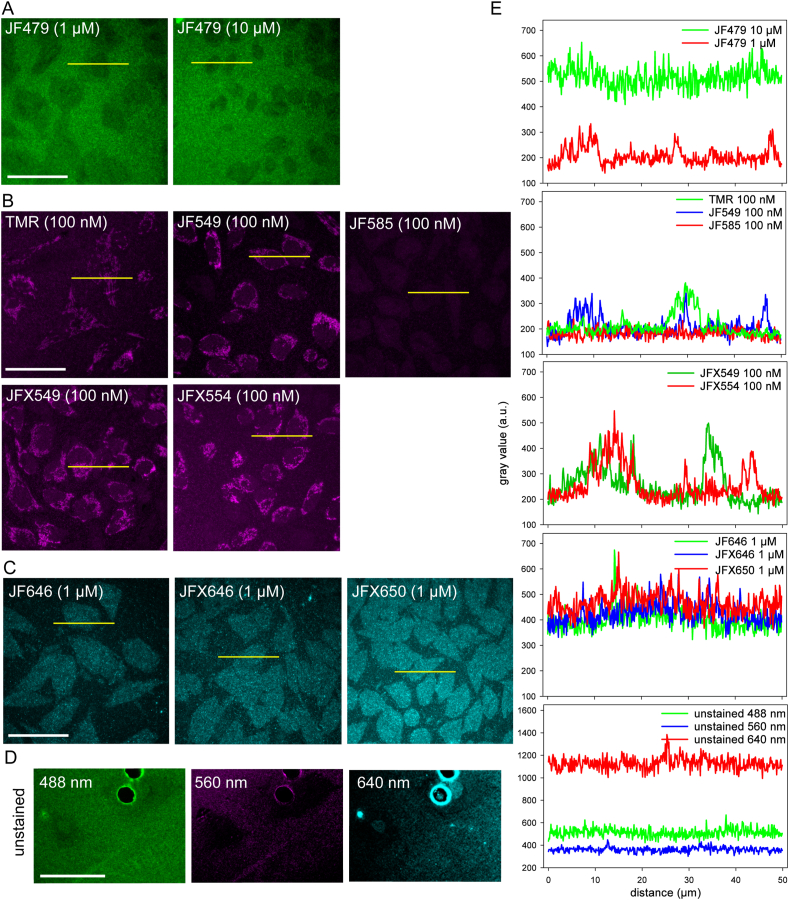
The red fluorescent dyes JF549, JFX549, JFX554, and TMR retain fluorescence after CE in EPON. HeLa cells stably expressing Tom20-HaloTag were stained with the indicated HTL conjugates with fluorescence emission in green (A), red (B), or far-red (C) spectra at the indicated concentrations for 30 min. Cells were fixed with glutaraldehyde and osmium tetroxide, and embedded in EPON resin at 60 °C for 48 h. Next, the samples were sectioned into 250 nm sections, and placed on a glass coverslip for imaging by widefield microscopy using an Olympus LSM FV3000 NLO. Analysis of the acquired images revealed loss of fluorescence for the green JF479 at both concentrations, for the fluorogenic red dye JF585, as well as for all tested far-red dyes, while fluorescence signals being reminiscent of mitochondria were detected for JF549, JFX549, JFX554, and TMR. The far-red dyes showed homogenous cellular fluorescence at dye concentration of 1 μM. D) Control HeLa Tom20-HaloTag cells without labeling with HTL conjugates were imaged in the green, red, and far-red channel and show background fluorescence of EPON, especially in the green channel. E) Quantification of signal intensities. Line scans of 50 μm (yellow lines in micrographs) were quantified for gray values expressed as arbitrary units (a. u.) over distance. Peaks in the plots represent either a specific fluorescence or parts of the speckled background resulting from EPON embedding (see e. g. peak from JF646). Scale bars: 50 μm. (For interpretation of the references to colour in this figure legend, the reader is referred to the Web version of this article.)

In a post-embedding CLEM approach, registration of fluorescence in the final sample is the critical and crucial feature. Therefore, we assessed the fluorescence retained directly on EPON sections. HeLa Tom20-HaloTag cells were stained with the respective dye and conventionally prepared for EM as described in Fig. 1. Sections of 250 nm thickness were placed on coverslips or 50 mesh grids and analyzed by LM. Since pre-tests could not detect any dye fluorescence in embedded cells stained with 100 nM JF479 and the green dye already displayed a low fluorescence signal in first LM (Fig. S3), JF479 was tested at 1 μM concentration, and with staining for 2 h rather than 30 min to enhance the fluorescence signal of JF479, and to counteracting the poor cell permeability of the dye. Yet, increased concentration and prolonged staining did not result in mitochondrial fluorescence signals in the embedded sample, although the fluorescence signal was strongly enhanced before sample preparation. Worth mentioning is the strong autofluorescence of EPON in the green channel, while autofluorescence was weaker in the red and far-red channels. To exploit further possibilities, the excitation LED power was increased to 100% and exposure time was set to 1 s, compared to 30% and 500 ms, respectively, used for the other dyes. Nevertheless, no fluorescence signals were observed for EPON sections of cells with Tom20-HaloTag-JF479 (Fig. 2A). Increasing the concentration to 10 μM, the upper reference value recommended by the supplier, also did not yield fluorescence signals of the dye in EPON sections (Fig. 2A).

In contrast, for HTL-ligands conjugated to red dyes JF549, JFX549, JFX554, or TMR, fluorescence signals representing the mitochondrial distribution were clearly detectable (Fig. 2B). The fluorogenic dye JF585 did not show any mitochondrial fluorescence and was comparable to the unstained control (Fig. 2B). Because JF585 already exhibited low fluorescence signals in LM, it was also examined at an increased concentration of 1 μM. Still, no specific fluorescence was observed on sections (Fig. S4).

The results of JF646, JFX646, and JFX650 also revealed lack of fluorescence retainment. Since initial tests with 100 nM of ligand did not result in detectable fluorescence on sections, concentration was increased to 1 μM. This led to higher homogeneous fluorescence of the entire cell, but still no specific fluorescence was observed despite a longer exposure time of 1 s and higher LED power of 100% (Fig. 2C). Line plots for all labeled samples are shown in panel Fig. 2E.

As mentioned above, apart from the fluorescence signal of the dyes, an uneven, speckled background signal (autofluorescence) was observed in the embedded samples. The background signal for extracellular EPON differed from the background signal within cells (Fig. 2A–D). In all channels, background fluorescence of EPON resin was brighter than cell-associated signals, as shown for an unlabeled control (Fig. 2D).

### 2.3 Quantification of fluorescence retainment in EPON

To evaluate fluorescence preservation of various fluorophores and their suitability, fluorescence intensities registered before and after EM sample preparation may be quantified and compared. However, in post-embedding CLEM the fluorescence finally retained in resin sections is the most important value. Also, it is not possible to compare the fluorescence signal of a whole cell in liquid buffer with an ultrathin cell section in EPON due to several reasons, like different Z, different medium, use of different LM settings and systems before and after EM sample preparation. Previously, other groups used an osmium tetroxide resistance assay [14,16,22], however the functionality of dyes is not only affected by the osmium step, but also by additional steps of EM sample preparation. We thus quantified fluorescence signal intensities of Janelia Fluor dyes and TMR in EPON sections of 250 nm thickness after CE sample preparation, from at least three independent experiments and at least 100 cells per experiment. For this, 250 nm sections were directly placed on a glass coverslip to facilitate LM. Registration of entire sections was conducted using widefield LM. Images were then analyzed with ImageJ following the algorithm described in Material and Methods.

**Fig. 3:**
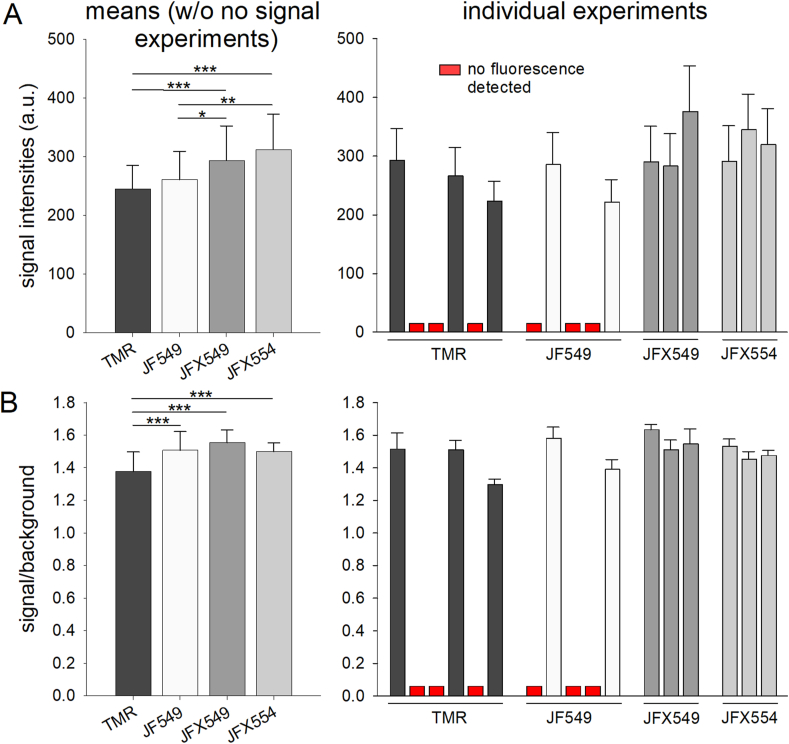
Comparison of fluorescence intensities and signal-to-background ratios of TMR, JF549, JFX549, and JFX554 in resin. HeLa cells stably expressing Tom20-HaloTag were stained with 100 nM of indicated HTL dye for 30 min. Cells were fixed and prepared as in Fig. 2. The average signal intensity and standard deviations were ascertained by the algorithm for the selection and the background of each acquired image. A) Mean signal intensities of all evaluated images. The standard deviation was computed by averaging the standard deviation determined by the algorithm for the signal intensity of the selection and is indicated by the whiskers. ii) displays the same as i) but subdivided into the individual experiments. B) Signal-to-background ratios (S/B) were computed by dividing the average signal intensity of the selection by the average signal intensity of the background for each image. Results of individual experiments were shown in the right panel, and indicate that labeling with HTL conjugated to TMR or JF549 frequently failed, while conjugates with JFX549 or JFX554 labeled in all cases. Only experiments resulting in detectable fluorescence were included in the calculation of means. Statistical analyses were performed by one-way ANOVA test and the Bonferroni *t*-test. Significances are indicated as follows: *, p < 0.05; **, p < 0.01; ***, p < 0.001.

Based on the results of our initial tests, only the dyes TMR, JF549, JFX549 and JFX554 were used for quantification. Our tests revealed that JFX554 and JFX549 were the best performing dyes, retaining nearly 20% more fluorescence in EPON sections compared to TMR (Fig. 3A). Interestingly, we also detected a significant difference between JF549 and the modified version JFX549 (Fig. 3A). Also note that TMR and JF549 did not show any fluorescence signals in 3 out of 6, and 3 out of 5 individual experiments, respectively, while retaining their fluorescence in the remaining experiments (Fig. 3). Apparently, TMR and JF549 are more susceptible to minor variation in EM sample preparation than other dyes, probably due to their structures and properties (Fig. 3). The signal-to-background (S/B) ratio was computed for the four dyes. The S/B ratio of JFX549 was slightly higher than that of JFX554 and JF549, yet not significant in difference. Comparison of S/B ratios of these dyes with that of TMR, which was the lowest, revealed a statistic significance at p < 0.001 (Fig. 3B).

To test if increased dye concentrations result in higher fluorescence intensities in resin, concentrations of 10 nM, 100 nM, or 1 μM or TMR or JFX554 were analyzed. Both dyes did not retain their fluorescence at 10 nM, which matches our observations of noticeable lower fluorescence signals at 10 nM in LM (data not shown). When comparing 100 nM to 1 μM TMR, a slight increase was found both in the fluorescence signal intensity and S/B ratio. JFX554, on the other hand, neither showed a statistically significant increase in the fluorescence signal, nor in the S/B ratio. Instead, the fluorescence signal rather slightly decreased (Fig. S5).

In conclusion, at 100 nM concentration and 30 min of labeling, JFX549 and JFX554 were the best-performing dyes under our conditions, not only displaying the brightest fluorescence signals in EPON sections after CE sample preparation, but also providing consistent and reproducible results.

### 2.4 In-resin CLEM

**Fig. 4:**
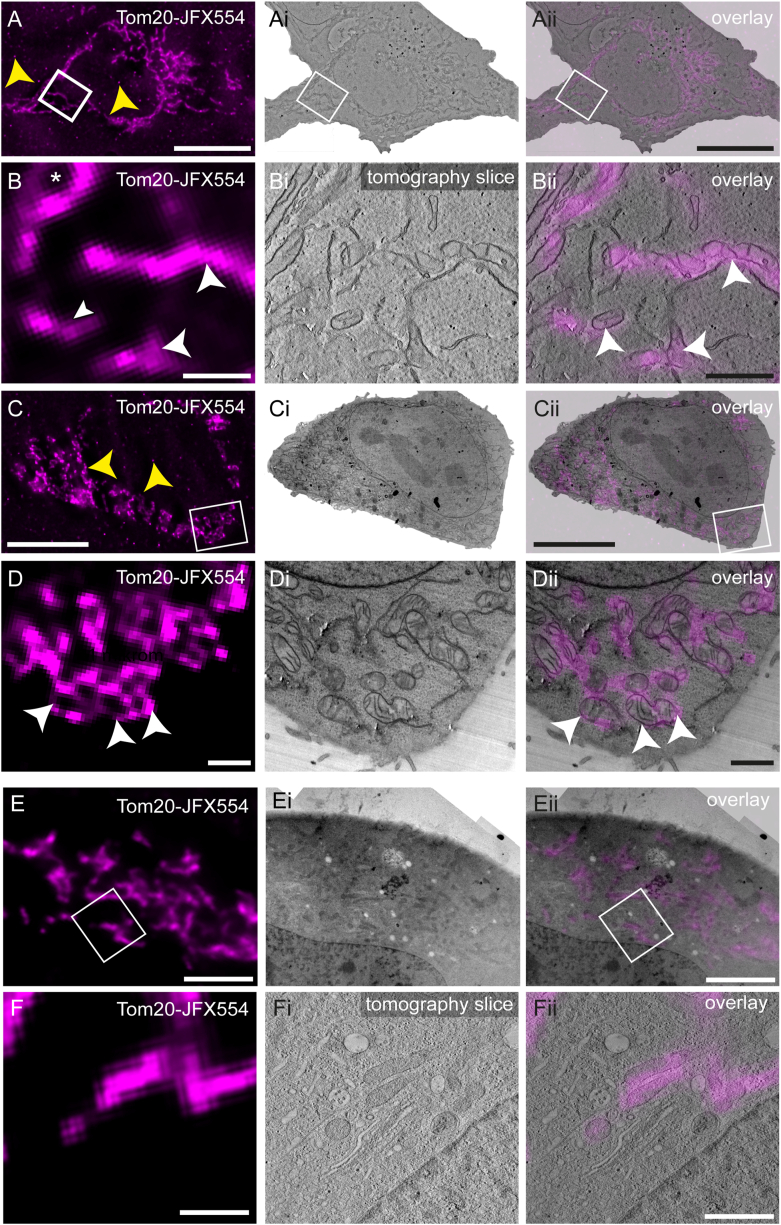
In-resin CLEM and tomography on 250 nm semithin or 100 nm thin sections after room temperature fixation or high-pressure freezing. HeLa cells stably expressing Tom20-HaloTag were stained with 100 nM HTL-JFX554 for 30 min and processed for in-resin CLEM via CE preparation (A–D) or HPF and FS (E, F). Sections of 250 nm thickness (**A, B, E, F**) were prepared for TEM tomography (**Bi,** see Movie 1 for tomogram; **Fi,** see Movie 2 for tomogram), and 100 nm sections (**C, D**) were imaged using a standard TEM setup (**Ci, Di**). In all cases, fluorescence was retained after sample preparation and allowed precise correlation of labeled mitochondria within the TEM images (**Aii, Bii, Cii, Dii, Eii, Fii**). Scale bars: A, Ai, Aii, C, Ci, Cii: 10 μm; E, Ei, Eii: 5 μm; B, Bi, Bii, D, Di, Dii, F, Fi, Fii: 1 μm.

We next examined the performance of JFX549 or JFX554 in representative CLEM workflows. HeLa cells stably expressing Tom20-HaloTag were stained with 100 nM JFX554 for 30 min, prepared for TEM including EPON CE, and sections of 100 nm or 250 nm thickness were prepared. In contrast to the experimental procedure for quantitative analysis of the dye, now the resin sections were subsequently transferred to formvar-coated 50 mesh grids, which were then placed between two glass coverslips with a drop of slightly alkaline PBS (pH 8.4) to improve fluorescence [35,36]. ROIs in sections were registered by LM, and subsequently analyzed by TEM after addition of gold fiducials and contrasting. Both semithin (250 nm) and ultrathin (100 nm) sections displayed well detectable fluorescence signals in LM (Fig. 4A, B, C, D). Nevertheless, a dot-like background, most likely resulting from the formvar coating and EPON resin, was clearly visible and might complicate detection of smaller, dot-like structures (Fig. 4A–C, yellow arrowheads). However, precise correlation with standard TEM images and even tomography data was readily possible, revealing single mitochondria in TEM positive for fluorescence signals (Fig. 4Bi, Bii, Di, Dii, Movie 1). Depending on the sectioning angle and thickness of the section even differentiation between the mitochondrial matrix and the inner and outer membrane system was possible (Fig. 4D, Di, Dii). To overcome the speckled background on sections, we tried to place the sections on mesh grids with a carbon film only (Plano, S160) as done by other groups to reduce background for methacrylate sections [12]. Unexpectedly, this resulted in severe deterioration of sample ultrastructure (Figs. S6A and B). This effect was independent from embedded biological material, since free EPON parts of sections also appeared affected (Fig. S6C) while carbon film without section was intact (Fig. S6D). To test whether different parameters during LM induce this deterioration, we placed sections from the same sample onto formvar-coated grids and subjected these to different steps of the LM workflow while also testing different pH of the PBS buffer. None of these parameters affected the ultrastructure on formvar-coated grids, suggesting that carbon-coated grids and EPON sections might be incompatible (Fig. S6E – Ev). LM of the liquid components of the EPON resin used does also not display the speckled background, indicating that the final polymerization might be the crucial step (data not shown). Furthermore, also EPON resin from other suppliers (i. e. Roth, Science Services) gave comparable background (data not shown).

Supplementary video related to this article can be found at doi:10.1016/j.heliyon.2024.e28055

The following is/are the supplementary data related to this article:

video 1 2 video 1

To clarify that the aforementioned red dyes are indeed suitable for in-resin CLEM, the workflow was conducted with TMR, JFX549, and in comparison JFX554. In order to test application to the fluorescence LM techniques, CLSM rather than widefield LM was used to register fluorescence signals. Here TMR, JFX549, and JFX554 yielded sufficient fluorescence signals in 250 nm sections, allowing precise correlation with TEM modalities (Fig. S7).

One important feature of LM is simultaneous visualization of several labeled subcellular structures within a biological sample. Since the green and far-red Janelia Fluor dyes did not work in our in-resin CLEM workflow while labeling mitochondria, we tested another far-red dye, i.e. Alexa Fluor 647, which was previously reported for CLEM approaches [37]. Alexa Fluor dyes are very versatile labels for LM due to their brightness and photostability. However, a major limitation is their cell impermeability. To test Alexa Fluor 647 for application in our workflow, the fluid tracer Dextran-Alexa Fluor 647 was used to pulse/chase HeLa Tom20-HaloTag cells, which were additionally stained with JFX554. Indeed, this approach resulted in well detectable JFX554-positive mitochondria and Alexa Fluor 647-positive vesicles, indicating potential use in dual-fluorescence in-resin CLEM (Fig. S8). Nevertheless, it remains to be determined if Alexa Fluor 647 is resistant in CE preparation comparable to e. g. JFX554, or if the high local concentration of endocytosed Dextran-Alexa Fluor 647 in vesicles prevented full quenching of fluorescence signals during sample preparation.

### 2.5 In-resin CLEM in combination with HPF and FS

High-pressure freezing (HPF) and freeze substitution (FS) are deployed for preserving ultrastructural features in a state that is closer to native than possible by CE preparation [11]. Since retention of TMR fluorescence after HPF and FS including osmium staining and EPON embedding was previously reporter [23], we tested if this also allies to JFX554. We strictly followed the protocol from Müller et al., but used HeLa Tom20-HaloTag cells in order to generate data that is comparable to our prior experiments. On 250 nm thin sections of these samples, fluorescence signals of 100 nM JFX544 were well detectable and allowed correlation with the corresponding cell in the TEM (Fig. 4E and F). Several cellular compartments such as nuclei, Golgi apparatus, or multivesicular bodies were well preserved, and mitochondria within the tomography volume were precisely correlated to JFX554 signals (Fig. 4E and F, Movie 2). These results indicate that JFX554 performs at least equal to TMR for in-resin CLEM after HPF and FS sample preparation.

Supplementary video related to this article can be found at doi:10.1016/j.heliyon.2024.e28055

The following is/are the supplementary data related to this article:

video 2 3 video 2

### 2.6 Rhodamine dyes represent versatile markers for identification of different cellular structures during in-resin CLEM

**Fig. 5:**
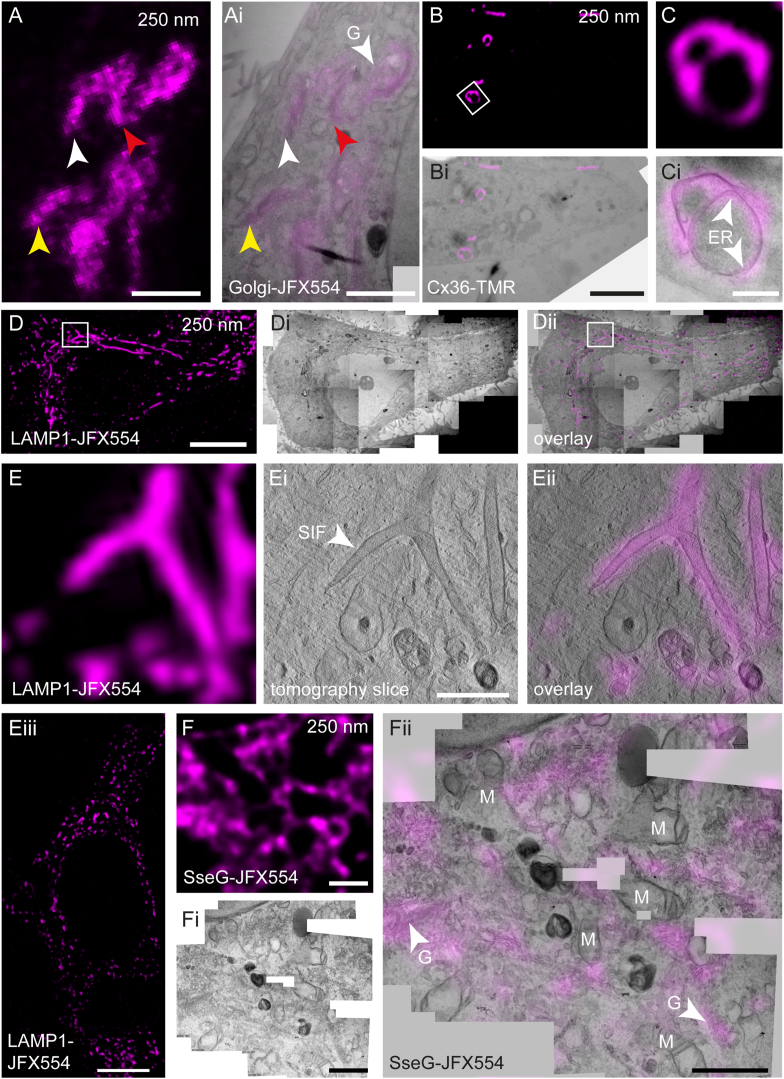
Various cellular structures can be addressed by in-resin CLEM. HeLa (**A**, **D**, **E**, **F**) or HEK (**B**, **C**) cells were transiently transfected for expression of Golgi-HaloTag (**A**), Connexin36-SNAP-tag (**B**, **C**), LAMP1-HaloTag (**D**, **E**), or the *Salmonella* Typhimurium effector protein SseG-HaloTag (**F**), stained with 100 nM HTL-JFX554 or HTL-TMR for 30 min, and conventionally prepared for EM. Sections of 250 nm (**A, B, C, D, E**) and 100 nm (**F**) thickness were prepared and imaged by LM (**A, B, C, D, E, F**). Fluorescence signals were retained allowing identification of labeled structures. Next, TEM tomography (**Ei**) or standard TEM imaging (**Ai, Bi, Ci, Di, Dii, Fi, Fii**) was performed, and precise correlations of the Golgi apparatus (**Ai, G**) or ER whorls (**Ci**) were obtained. HeLa cells expressing LAMP1-HaloTag were infected with *S.* Typhimurium, and 8 h post infection cells were stained with HTL-JFX554 and conventionally prepared for EM (**D, E,** see Movie 3 for tomogram). Tubular LAMP1-positive structures induced by *Salmonella* are clearly visible. A branching SIF can be observed in LM and TEM tomography (**E, Ei, Eii**). Correlation reveals the tubular architecture of LAMP1-positive membranes (**Eii**). In an uninfected control cell, the endo-lysosomal system is not modified (Eiii). After expression in HeLa cells, SseG localizes at various compartments of the Golgi apparatus (**F, Fi, Fii**). Scale bars: A, Ai: 2 μm; B, Bi: 5 μm; C, Ci: 500 nm; D, Di, Dii, Eiii: 10 μm; E, Ei, Eii, F, Fi, Fii: 1 μm.

To underline the versatility and general applicability of the CE in-resin CLEM workflow described here, different cellular structures were stained with 100 nM JFX554 or TMR and visualized via in-resin CLEM (Fig. 5). LM on 250 nm semithin sections of HeLa cells expressing a Golgi-HaloTag marker revealed different Golgi stacks in a cell closely correlating with the underlying ultrastructure (Fig. 5A–Ai). In a HEK cell line expressing Connexin36 (Cx36) tagged with SNAP-tag, extensive membrane whorls were detected in LM (Fig. 5B and C). Correlation with the underlying ultrastructure rapidly allowed identification of these structures and suggested that these originate from the endoplasmic reticulum (ER) (Fig. 5Bi, Ci), which is in line with recently published data [38].

To validate that also further biological questions can be tackled with the in-resin CLEM approach, we set out to recapitulate CLEM results for the endosomal remodeling in mammalian infected by *Salmonella enterica* serovar Typhimurium (STM) [9]. For this, HeLa cells expressing LAMP1-HaloTag were infected with STM, at 8 h post infection the cells were stained with 100 nM JFX554 and prepared for in-resin CLEM. The fluorescence signal of LAMP1-positive *Salmonella*-induced filaments (SIFs) and also other endosomal structures was clearly visible in 250 nm semithin EPON sections (Fig. 5D). The accurate correlation allowed us to precisely identify a branching filament and to visualize the complex membrane architecture by TEM tomography (Fig. 5E, Ei, Eii, Movie 3). In-resin LM of LAMP1-HaloTag of non-infected HeLa cells indicated LAMP1-HaloTag signals with typical distribution of late endosomes/lysosomes but absence of SIF (Fig. 5Eiii).

Supplementary video related to this article can be found at doi:10.1016/j.heliyon.2024.e28055

The following is/are the supplementary data related to this article:

video 3 4 video 3

To further benchmark the system, we transfected HeLa cells for expression of STM effector protein SseG conjugated with HaloTag, and labeled the fusion protein with 100 nM JFX554. After sample preparation, the fluorescence signal was still detectable in resin (Fig. 5F) which greatly facilitated correlation of the effector protein with parts of the Golgi apparatus (Fig. 5Fii).

These results clearly demonstrate that the rhodamine dyes TMR, JFX549 and JFX554 are suitable markers for targeting of different cellular structures on 250 nm semithin EPON sections, while keeping the sample preparation extremely simple. Thus, high precision on-section CLEM can be performed in fast and convenient manner. Adoption of such CE workflow by EM labs is easily possible and does not require additional equipment or chemicals.

### 2.7 In-resin Lattice Light-Sheet Structured Illumination Microscopy utilizing JFX554

**Fig. 6:**
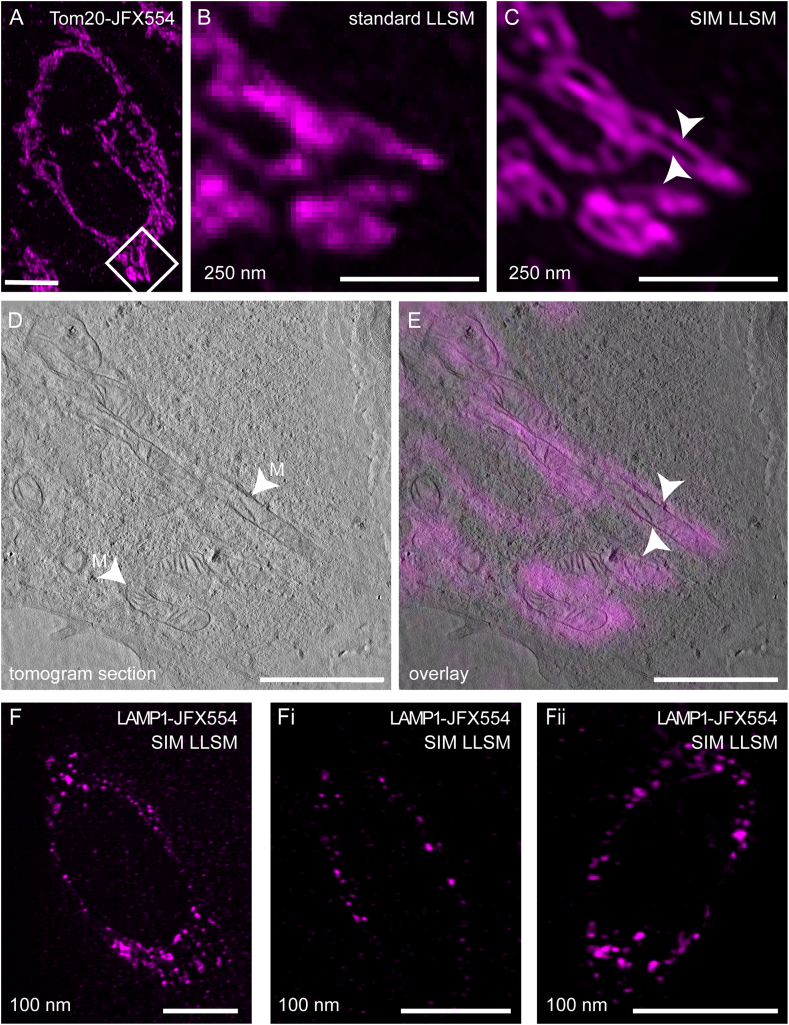
Application of HaloTag ligands with Janelia Fluor dyes for in-resin super-resolution microscopy and CLEM. HeLa cells stably expressing Tom20-HaloTag were labeled with 100 nM HTL-JFX554 for 30 min and conventionally prepared for EM. 250 nm semithin sections were prepared and subjected to Lattice Light-Sheet Microscopy (LLSM) in combination with Structured Illumination Microscopy (SIM, **A, B, C**). The comparison of images obtained by LLSM (**B**) or SIM (**C**) modalities indicates the improved spatial resolution of SIM. After performing TEM tomography (**D,** see Movie 4 for tomogram), the super-resolved LM image was precisely correlated with mitochondrial membranes (**E,** M). LLSM-SIM was also possible on 100 nm thin sections of HeLa cells stably transfected with LAMP1-HaloTag and labeled with 100 nM JFX554 for 30 min (**F, Fi, Fii**). Scale bars: A: 5 μm; B, C, D, E: 2 μm; F, Fi, Fii: 10 μm.

A main problem of CLEM workflows is the large gap in resolution between LM and EM. To overcome this issue, researchers started to integrate super-resolution microscopy (SRM) into CLEM workflows. To evaluate the possibility of using red-fluorescent Janelia Fluor dyes for an in-resin SRM workflow, we deposited 250 nm EPON sections on 50 mesh grids and registered FM modalities using Lattice Light-Sheet Microscopy (LLSM) on a set-up also capable of performing Structured Illumination Microscopy (SIM). We selected LLSM because other setups such as Total Internal Reflection Microscopy (TIRF) require very plane surfaces such as glass coverslips to obtain high quality images. Due to the fact that LLSM does not require completely plane surfaces, we predicted it to be more suited to image the varying surface of the EPON section on the grid. To test this, we labeled HeLa Tom20-HaloTag cells with 100 nM JFX554, conventionally prepared EM sections of 250 nm thickness, which were deposited onto 50 mesh formvar-coated EM grids and inserted into a custom-made holder for LLSM. With this approach we were able to successfully record in-resin SIM data, which, in comparison to standard LLSM imaging, benefit from increased resolution, allowing us to clearly depict the gap between membranes and matrix of mitochondria (Fig. 6A, B, C, white arrowheads). Finally, retrieval of the same cell in TEM and a tomogram facilitated a precise correlation between both imaging modalities (Fig. 6D and E, Movie 4). Furthermore, LLSM-SIM was also possible on 100 nm thin sections of HeLa cells stably transfected with LAMP1-HaloTag and labeled with 100 nM JFX554 for 30 min (Fig. 6F).

Supplementary video related to this article can be found at doi:10.1016/j.heliyon.2024.e28055

The following is/are the supplementary data related to this article:

video 4 5 video 4

### 2.8 Dual fluorescence in-resin CLEM

**Fig. 7:**
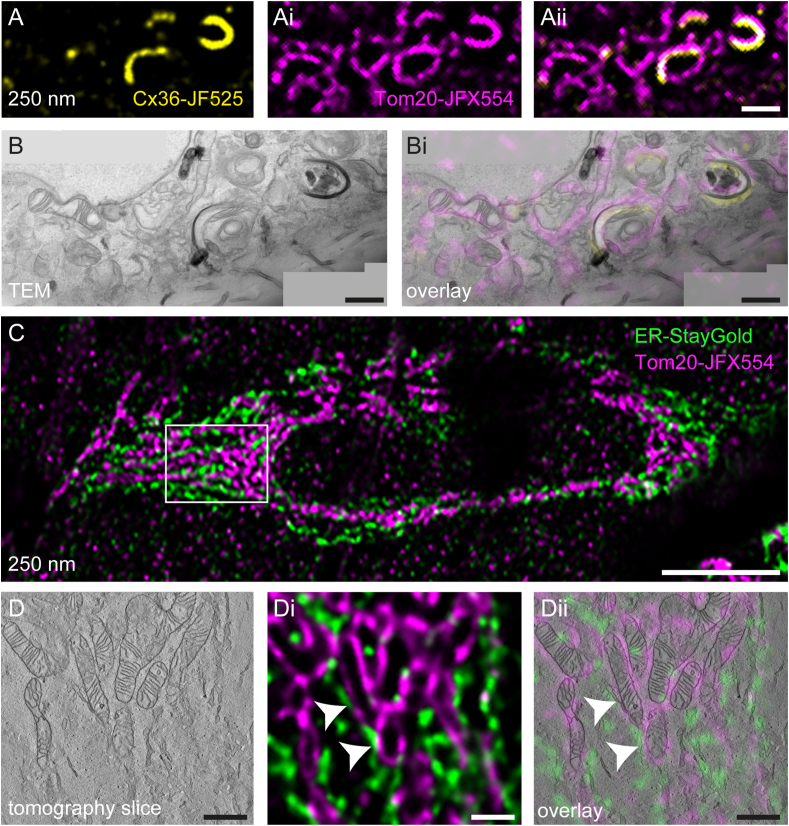
Dual-fluorescence in-resin CLEM using combinations of HaloTag and SNAP-tag, or HaloTag and fluorescent protein StayGold. HeLa cells stably expressing Tom20-HaloTag were transiently transfected for expression of Connexin36-SNAP-tag (**A, B)** or ER-StayGold (**C, D**), labeled with 100 nM HTL-JFX554 (magenta) and SNAP-tag ligand-JF525 (yellow) for 30 min and conventionally prepared for EM. 250 nm semithin sections were prepared and subjected to dual-fluorescence LM (**A, Bi, C, Ci, Cii**). Fluorescence signals of JF525 and JFX554 were retained in resin, allowing simultaneous detection of ER whorls and mitochondria and their correlation (**A, Ai, Aii, B, Bi)**. Fluorescence signals of StayGold (green) and JFX554 (magenta) were retained in resin, allowing correlation of ER and mitochondria within the tomographic volume towards detection of membrane contact sites (**C, D, Di, Dii,** white arrowheads, Movie 5). Scale bars: A, Ai, Aii, B, Bi, D, Di, Dii: 1 μm; C: 10 μm. (For interpretation of the references to colour in this figure legend, the reader is referred to the Web version of this article.)

Simultaneous labeling and visualization of multiple cellular structures tremendously extends the application of CLEM workflows. One potential dual-fluorescence was shown in Fig. S8, but his approach relied on endocytic uptake of a tracer with one of the dyes. For a more versatile approach, independent labeling of two cellular structures is preferred. Since the green dyes tested in this work did not yield in-resin fluorescence, we considered JF525 as another candidate for dual-fluorescence experiments, since the emission and excitation wavelengths of this dye are slightly shifted into the yellow regime. We transfected Tom20-HaloTag expressing HeLa cells for transient expression of Cx36-SNAP-tag, expected to localize to ER membranes and to induce ER whorls upon overexpression [38]. Simultaneous staining of SNAP-tag and HaloTag with 100 nM SNAP-ligand JF525 and HTL JFX554, respectively, was performed. Intriguingly, JF525 retained fluorescence in-resin, allowing the localization of ER whorls on EPON sections (Fig. 7A). Correlation of ER whorls and mitochondria was successful with this set of dyes (Fig. 7B, Bi). However, in combination with JFX554, we observed bleed-through into the red channel (Fig. 7Ai, Aii), probably limiting application of JF525 and JFX554 in dual-fluorescence experiments.

To further investigate the possibility of dual-fluorescence detection, we considered fluorescent proteins for in-resin fluorescence as reported in various publications [14,16,39]. Since we already focused on very bright and photostable dyes in this study, we considered that fluorescent proteins with comparable properties might be suitable for in-resin CLEM. However, the previously reported EM-optimized fluorescent proteins did not retain sufficient fluorescence in our workflow in combination with Janelia Fluor Dyes (data not shown). As an alternative, we tested the recently published green fluorescent protein StayGold [40] for potential applicability in our in-resin CLEM approach. StayGold was shown to be very photostable and bright in FM applications, and therefore being a very promising candidate. We showed that in cells transfected for expression of ER-StayGold, fluorescence was well visible on EPON sections (Fig. 7C). The combination with Tom20-HaloTag-JFX554 allowed dual-fluorescence in-resin CLEM of ER and mitochondria (Fig. 7D, Di, Dii). With this, for example, membrane-contact sites can be readily detected (Fig. 7Di, Dii).

In conclusion, we identified StayGold as fluorescent protein resistant to CE preparation, as well as JF525 as another dye for SLE ligand retaining fluorescence in-resin for use in dual-fluorescence CLEM.

## 3 Discussion

Our study examined the performance of selected HTL-conjugated dyes, namely JF479, JF549, JF525, JFX549, JFX554, JF585, TMR, JF646, JFX646, JFX650, in a post-embedding in-resin CLEM approach. Initial LM revealed that labeling HeLa Tom20-HaloTag cells with 100 nM of each dye for 30 min before fixation provided sufficient fluorescence signals matching the distribution of mitochondria for most dyes. Only JF479 and JF585 presented an exception, as their fluorescence signal was barely visible in LM. The various fluorophores used here have distinct properties regarding membrane permeability, labeling kinetics, excitation and emission spectra, and may require individual optimization to perform for in-resin CLEM. Here we rather opted to identify fluorophores that can be easily integrated in CE sample preparations, performed with standard microscopy equipment and settings, and in reasonable concentrations. By applying the same labeling conditions for in-resin CLEM studies, we observed in-resin fluorescence only in cells labeled with TMR, JF525, JF549, JFX549 and JFX554. All samples were fixed with osmium tetroxide and embedded in EPON. In-resin fluorescence was detectable using the HaloTag or SNAP-tag after labeling structures such as ER, Golgi apparatus, mitochondria, or the *Salmonella* effector protein SseG. Our results demonstrate that a subset of fluorophores retains fluorescence in resin, allowing CLEM applications.

With respect to TMR, the concentration of 100 nM for 30 min used here was considerably lower than in previous work by Los et al. [41] in LM (5 μM TMR for 15–60 min), Perkovic et al. [42] in Lowicryl sections after HPF and FS (50 μM TMR for 30 min), or by Müller et al. [23] in EPON sections chemically fixed and stained with osmium tetroxide (0.6 μM overnight or 6 μM for 1 h using TMR-Star). Using lower fluorophore concentration brings the advantage of lower cell toxicity, reduced preparation costs, and the reduction of washing steps needed to remove excess unbound label. In the quantitative comparison of the in-resin fluorescence of different dyes, we found JFX554 and JFX549 to be the best performing among all tested dyes in terms of signal intensity and S/B ratio. Our findings complement the results of Müller et al. [23], who successfully used SNAP- and CLIP-tag in combination with the respective TMR-ligand to label structures such as insulin or LifeAct in post-embedding CLEM experiments, highlighting the suitability of all three commonly used SLE for CLEM.

Our tests for in-resin fluorescence of the green JF479, the red fluorogenic JF585 and the far-red dyes JF646, JFX646 and JFX650, included higher concentrations of 1 μM for each of these dyes, and even 10 μM for JF479 were tested. Previous studies used these JF dyes for LM of labeled proteins conjugated to HaloTag at concentrations between 100 and 500 nM [[28], [29], [30], [31],^43^], sometimes up to 1 μM [33,34]. However, in our application the increased concentrations did not improve the results on EPON sections.

Why exactly certain fluorophores perform in our workflow and other fail is unclear. The chemical structure of the fluorophores provides some indications for the different outcomes. All fluorophores retaining fluorescence in-resin possess an oxygen atom within the xanthene structure. The retainment also seems to be, at least to some degree, independent from the excitation and emission wavelength, since also JF525 retained fluorescence in-resin. In contrast, this atom is replaced by silicon in JF646, JFX646 and JFX650, by nitrogen in JF479, or by carbon in JF585. Thus, this oxygen atom may stabilize the fluorophore during EM sample preparation. Alternatively, the difference between the fluorophores could arise solely from the silicon and the 3,3-difluoroazetidines as these two features apply only to the far-red dyes and to JF479 and JF585, which lost in-resin fluorescence. In order to verify this hypothesis at least with respect to the 3,3-difluoroazetidines, the antecedents of JF479 and JF585, namely JF502 and JF608 [25,27], could be tested as they comprise nitrogen and carbon atoms, respectively, but not the 3,3-difluoroazetidines.

To identify and optimize fluorophores for in-resin CLEM, future work should systematically investigate the effect of various modifications of the JF dyes chemical structures on their resistance to CE preparation. In this regard, dyes may be engineered which are especially resistant and useful for in-resin CLEM similar to optimization of fluorescent proteins (FP) for EM such as mEosEM [16]. Moreover, optimized variants of SLE with increased fluorescence intensity of bound dyes should be tested for in-resin fluorescence retainment [44].

A recent approach to simplify post-embedding CLEM deploys specifically engineered FP which can, to some degree, tolerate the quenching properties of CE protocol [14,16,21,22]. However, many of these FP apparently retain only comparably weak fluorescence signals and low S/B ratio after EPON embedding. For sufficient preservation of FP fluorescence, reduced osmium tetroxide concentrations and/or incubation times such as 10 min were applied. This, however might severely reduce ultrastructural details in TEM modalities. Furthermore, in these publications, in-resin fluorescence was always registered on sections being on glass coverslips. This format allows SEM imaging, but is not compatible with 2D or 3D TEM imaging, or requires the removal of the glass from the resin sections and support film via hazardous hydrofluoric acid etching [14,16,21,22,24]. In contrast to all these adaptations which were made to potentially increase fluorescence preservation, we identified StayGold as EM-resistant fluorescent protein. StayGold is a promising marker for in-resin CLEM, since it does not need special adjustments to the workflow to function in-resin but can easily integrated into CE preparation. One potential drawback of StayGold, its high tendency to dimerize, has recently been addressed by generation of monomeric variants [45,46].

The importance of testing the performance of various dyes in on-section CLEM workflows with CE sample preparation is supported by recent publications in this field [23,24,37]. So far, only Müller et al. worked with cell-permeable dyes in such setup, and reported successful application of TMR and 505-Star coupled to ligands of HaloTag, SNAP-tag or CLIP-tag. However, the protocols used in Müller et al. [23] calls for strongly reduced osmium concentrations of 0.1%, and did not deploy potassium hexacyanoferrate as a further contrast enhancer, as done in our approach. Since their study was not focused on systematically or quantitatively testing the performance of a larger set of dyes, we here aimed to tackle this point. Interestingly, the group showed that 505-Star within insulin granules survived sample preparation. We speculate that either large amounts of dye accumulated in these insulin granules and were not completely quenched during the sample preparation, or that 505-Star represents a promising alternative to the green dyes tested in our work. This could conceivably be the case, because the structure of 505-Star contains an oxygen atom within the xanthene, comparable to JF525 or JFX554, and following our earlier hypothesis, possibly making it more resistant to EM sample preparation. Sanada et al. [24] and Tanida et al. [37] investigated various cell-impermeable dyes for their performance after CE preparation and and found that red dyes such as DyLight549, iFluor546 or HiLyte-555 performed best, while detection of Alexa Fluor 647 was also still possible in resin. Our results with cell-permeable dyes complement these findings, as we also identified red dyes as best-performing, while Alexa Fluor 647 also retained fluorescence. A great downside of the protocol employed in their work [24,37] is the need for cell permeabilization to allow dyes to access intracellular targets. Permeabilization strongly affects ultrastructural quality of EM samples [47].

A comparable, well-established method to preserve the fluorescence within biological samples consists of embedding high-pressure frozen (HPF) samples in Lowicryl resins after freeze substitution (FS) with low concentrations of uranyl acetate [12,13,42,48]. For this, no special dyes or SLE are needed, but standard fluorescent proteins like GFP or mCherry can be used. For some JF dyes, we also demonstrated the applicability in HPF and FS workflows with EPON embedding, and we anticipate that dyes will also perform at least as equally well in hydrophilic resins, or at better labeling conditions. However, such workflows are challenging, require advanced and expensive equipment and osmium tetroxide cannot be used to enhance contrast for EM. Furthermore, the best ultrastructural preservation is most often obtained in EPON resin [49]. Therefore, the workflow described here provides an easy and cheap alternative for in-resin CLEM which can be incorporated by every EM facility without need for additional equipment or fundamental changes in sample preparation protocols to ensure fluorescence preservation. In addition, the here tested JF dyes allow in-resin SRM improving the correlation of LM and EM modalities [50].

In conclusion, our analyses provide a first systematic basis to further characterize und understand the behavior of fluorophores after CE preparation for TEM. The red dyes JFX554 and JFX549 represent the most promising candidates for use in CE in-resin CLEM approaches. We hypothesize that specifically the oxygen atom within the xanthene structure of the various dyes plays an important role in withstanding the harsh conditions of the protocol, and in retaining fluorescent properties to sufficient degree. Overall, the workflow described here has the advantages of being fast, easy to use, only requiring low concentrations of dyes, and being easily integrated into standard workflows of imaging facilities.

## 4 Materials and methods

### 4.1 Cell lines and cell culture conditions

The epithelial cell line HeLa (American Type Culture Collection, ATCC no. CCL-2) and derivatives (Table 2) were cultured in Dulbecco's modified Eagle medium (DMEM) containing 4.5 g/l glucose, 4 mM stable glutamine and sodium pyruvate (Biochrom), and 10% inactivated fetal calf serum (iFCS) (Sigma-Aldrich) at 37 °C in an atmosphere containing 5% CO_2_ and 90% humidity. HEK-293 F T (humane embryonic kidney, Invitrogen no. R700-07) cells were cultured the same way. For CLEM, cells were seeded into 8-well μ-slides with polymer bottom (ibidi) with (Art. 80,826-G500) or without (Art. 80,806) an engraved coordinate system. Cell culture was performed to achieve about 50–60% confluency on the day of the experiment.

### 4.2 Transfection of cells

HeLa or HEK cells were cultured for at least one day and transfected using FuGENE HD reagent (Promega) according to manufacturer's instruction. Plasmids used for transfection are listed in Table 3. Briefly, 0.5–2 μg of plasmid DNA were dissolved in 25–100 μl DMEM without iFCS and mixed with 1–4 μl FuGENE reagent (ratio of 1:2 for DNA to FuGENE). After 10 min incubation at room temperature (RT) the transfection mix was added to the cells in DMEM with 10% iFCS for at least 18 h. Before infection or staining, cells were provided with fresh medium without transfection mix.

### 4.3 Generation of stably transfected HeLa cells

Plasmid p5664 for expression of LAMP1-HaloTag was regenerated by Gibson assembly using lentiviral vector pLX304 as backbone. HeLa cells stably expressing LAMP1-HaloTag were generated using 3rd generation lentiviral vectors (The RNAi Consortium, 2015) and subsequent fluorescence-activated cell sorting (FACS). For the generation of lentiviruses, HEK 293 F T cells (3.8 × 10^5^ cells × ml^−1^, Invitrogen R700-07) were seeded in 10 cm tissue culture plates (TPP) with 10 mL antibiotic-free growth medium (high glucose DMEM + 10% iFCS). After 24 h cells reached 70%–80% confluency and were transfected with lentiviral packaging plasmid (9 μg pSPAX2), envelope plasmid (0.9 μg pMD2.G), and p5664 for LAMP1-HaloTag (9 μg) using 36 μl Opti-MEM (Fisher Scientific) and 54 μl FuGENE HD transfection reagent (Promega). The transfection mix was incubated for 30 min at RT before dropwise addition to cells. Cells were incubated for at least 18 h, then medium was changed to 15 ml growth medium containing 30% iFCS. After 24 and 48 h, medium containing lentiviruses was harvested and centrifuged at 350×*g* for 5 min to pellet any residual packaging cells. Supernatant obtained was stored in sterile polypropylene tubes at −80 °C. Half of the harvested supernatant was filtered using fast flow & low binding filters (Merck Millipore).

For lentiviral transfection, HeLa cells (1 × 10^5^ cells x ml^−1^) were seeded in 24-well plates (TPP) and incubated for 24 h before 8 μg x ml^−1^ Polybrene (Sigma) was added to the cells. After 48 h the filtered supernatant containing lentiviruses was added to the HeLa cells. After 48 h growth medium was exchanged to DMEM containing 10 μg x ml^−1^ Blasticidin (Invitrogen) and cells were incubated for 72 h. Then medium was changed to DMEM without antibiotics. After 24 h cells were detached for cultivation in tissue culture flasks (TPP) to obtain sufficient cell numbers for FACS.

For FACS, transfected HeLa cells were incubated with growth medium containing 100 nM HTL-TMR (Promega) for 30 min, then rinsed thrice with PBS and further incubated with medium for 30 min. Stained cells were detached from tissue culture flasks and resuspended at 1 × 10^7^ cells × ml^−1^. A 100 μm cell strainer (BD Falcon) was used to remove cell aggregates prior to FACS. Cells were sorted for red TMR fluorescence by on SH800S FACS (Sony) and a mixed population of cells with different LAMP1-HaloTag expression levels was obtained. This population was cultured again in tissue culture flasks before a second round was conducted. A homogenous population was obtained and analyzed regarding HTL-TMR intensity, cell division, and phenotypes of intracellular *Salmonella* prior to use in further experiments.

### 4.4 Infection of cells

For infection of HeLa cells, *Salmonella enterica* serovar Typhimurium NCTC12023 strains were grown in LB broth with appropriate antibiotics overnight (O/N), diluted 1:31 in fresh LB with antibiotics and subcultured for 3.5 h at 37 °C. The infection of HeLa cells was performed at various multiplicities of infection (MOI) for 25 min by directly adding a suitable amount of *Salmonella* subculture to the cells. Subsequently, cells were washed thrice with PBS and incubated for 1 h with DMEM containing 100 μg/ml gentamicin (Applichem) to kill extracellular bacteria. Finally, the medium was replaced by DMEM containing 10 μg/ml gentamicin for the rest of the experiment.

### 4.5 Staining of cells with fluorescently labeled ligands

Dyes coupled to their respective ligands as listed in Table 1 were dissolved in DMSO, diluted in PBS or cell culture medium, and directly added to cell culture medium to yield the appropriate final concentrations. After 30 min of incubation time at 37 °C, 5% CO_2_, 90% humidity, if not stated otherwise, stained cells were washed with PBS 3–5 x for 1 min to remove unbound dye, and subsequently fixed.

### 4.6 Fixation of cells for pre-embedding light microscopy

For pre-embedding light microscopy, 37 °C pre-warmed fixative containing 3% PFA and 0.1–0.2% GA in 0.1 M sodium cacodylate buffer (pH 7.2) was added to cells and incubated for 30 min at RT. Subsequently, samples were washed 3–5x for 1 min with 0.1 M sodium cacodylate buffer and either directly imaged or stored in the dark at 4 °C.

### 4.7 EM sample preparation

During the entire procedure, samples were kept in the dark as far as possible. First, samples were fixed with either pre-warmed (living cells) or 4 °C cold (fixed cells for LM) 2.5% GA in 0.1 M sodium cacodylate buffer (pH 7.2) for 1 h. Living cells were kept for 15 min at 37 °C in fixative and were then transferred to RT for further incubation. Then, samples were placed on ice for another 30 min before washing with 0.1 M sodium cacodylate buffer (pH 7.2) thrice for 1 min. Samples were post-fixed for 30 min with 1% OsO_4_ + 1.5% K_4_[Fe(CN)_6_] in 0.1 M sodium cacodylate buffer (pH 7.2). Subsequently, samples were washed 5x for 1 min with 0.1 M sodium cacodylate buffer (pH 7.2) and dehydrated. For dehydration, samples were successively incubated in 30%, 50% 70%, 80%, 90%, 100% ethanol and 100% anhydrous ethanol for 7 min each on ice and allowed to reach RT during the last step. After two incubation steps in 100% anhydrous acetone for 7 min each, samples were infiltrated on a shaker (50–60 rpm) with 25%, 50%, and 75% EPON (Sigma-Aldrich) diluted in anhydrous acetone for 1 h each with closed lids. Lids were removed from 8-well μ-slides and the samples were kept on the shaker in 100% EPON O/N. EPON was exchanged the next morning and again after 6 h. Thereafter polymerization was conducted at 60 °C for 48 h. The polymerized EPON blocks were sawed out from the 8-well μ-slides together with the polymer bottom which was removed by placing the bottom of the EPON blocks in toluol (Roth) and frequently wiping it with a paper towel. Each EPON block was sawed into 4 smaller blocks, trimmed to a trapezoid and sectioned into 100 nm or 250 nm sections using an ultramicrotome (Leica ULTRACUT EM UC7RT or UC7cryo). For quantitative comparison experiments the sections were placed on a glass coverslip previously washed in 100% ethanol. For correlative experiments the sections were transferred to commercial carbon-coated (Plano, S160), or formvar-coated (custom-made) EM grids.

### 4.8 Pre-embedding light microscopy

Light microscopy prior to EM sample preparation was either conducted at a Leica CLSM SP5 equipped with HC PL FL 10x (NA 0.3), HCX PL APO CS 40x (NA 0.75–1.25, oil immersion) and HCX PL APO CS 100x (NA 0.7–1.4, oil immersion) objectives or at a Zeiss Cell Observer SD equipped with Alpha Plan-Apochromat 63x (NA 1.46, oil immersion) and Plan-Apochromat 10x (NA 0.45, air) objectives. Samples were screened for ROIs and subsequently high magnification Z-stacks (100x or 63x objectives) and low magnification (10x objectives) overview images revealing the 8-well μ-slide coordinate system for relocation were acquired.

### 4.9 Light microscopy of EPON sections (in-resin LM)

For in-resin LM, a 10 μl drop of PBS (pH 8.4) was added respectively onto two 25 mm glass coverslips. The EM grid carrying the resin sections was placed on top of one of the PBS drops and subsequently sandwiched with the remaining coverslip. The assembly was transferred into a custom-made holder and imaged with the sections facing the objective lens using an Olympus FV-3000 with settings listed in Table 5. The microscope was either operated as a CLSM or as a widefield system as indicated in figure legends and was equipped with a sCMOS camera (ORCA‐Flash 4.0, Hamamatsu, Japan). Using appropriate filter/detector settings for the specific dyes, Z-stacks (step-size 200–400 nm) were acquired with a 60x oil immersion lens (PLAPON-SC NA 1.4). Subsequently, overview images of the grid utilizing the 10x air objective (UPL SAPO NA 0.4) facilitating correlation later on in the TEM were recorded. Frequently used exposure times for visualizing the different dyes ranged between 500 ms–1000 ms. After imaging, the grid was removed from the coverslip sandwich, washed thrice in distilled water, dried by touching a filter paper and stored in the dark.

**Table 5:** Settings for quantitative image acquisition at Olympus LSM FV3000 NLO.

Dye	Channel	Excitation wavelength	LED	Exposure time	Condensor	Filter
JF479	GFP	488 nm	100%	1 s	No	409/493/573/652 (LED)
JF549, JFX549, JFX554, JF585, TMR	TMR	560 nm	30%	500 m s	No	409/493/573/652 (LED)
JF646, JFX646, JFX650, Dextran-Alexa Fluor® 647	Cy5	640 nm	100%	1 s	No	409/493/573/652 (LED)

Lattice Light-Sheet Microscopy (LLSM) and Lattice Light-Sheet Microscopy Structured Illumination Microscopy (LLSM-SIM) were performed using custom-system based on the original design by the Eric Betzig group [51]. EM grids carrying a section were inserted into a custom-built sample holder that was mounted onto a piezo stage for sample scan imaging. This ensured that the sample was inserted at the correct position inside a sample bath, which contained PBS, pH 7.4 at 25 °C. For standard LLSM, stacks were acquired in sample-scan mode moving cells through a fixed light-sheet with a step size of 400 nm, which is equivalent to ∼216 nm slicing with respect to the Z-axis, when considering the sample scan angle of 32.8°. A dithered square lattice pattern was used, generated by multiple Bessel beams using an inner and outer numerical aperture of the excitation objective of 0.48 and 0.55, respectively. JFX554 was excited using a 561 nm laser (2RU-VFL-P-2000-561-B1R; MPB Communications Inc.). The final lattice light-sheet was generated by a water-dipping excitation objective (54–10–7 @488–910 nm, NA 0.66, Special Optics, NJ, USA), while emitted photons were collected by a water dipping detection objective (CFI Apo LWD 25XW, NA 1.1, Nikon, Tokyo, Japan). Emission was filtered with a 593/46 BrightLine HC bandpass filter (Semrock) and imaged on a sCMOS camera (ORCA‐Fusion, Hamamatsu, Japan) with a final pixel size of 103.5 nm and 50 ms exposure time. Raw data were further processed by using an open-source LLSM post-processing utility called LLSpy (https://github.com/tlambert03/LLSpy) for deskewing and deconvolution. Deconvolution was performed by using an experimental point spread function, recorded from 100 nm sized FluoSpheres™ (Art. No. F8801, ThermoFisher Scientific) and is based on the Richardson–Lucy algorithm using 10 iterations. Structured Illumination Microscopy (SIM) was performed with a hexagonal lattice light-sheet in accordance to Chen et al. [51]. Reconstruction was done with an implementation of the algorithm for 3D-SIM [52], which was previously adapted for Bessel beam structured plane illumination microscopy [53,54]. The OTF used was calculated from an experimentally measured PSF recorded in SIM mode from the same 100 nm sized FluoSpheres™.

### 4.10 Transmission electron microscopy (TEM)

Resin sections on EM grids contrasted with 3% uranyl acetate for 30 min and 2% lead citrate for 20 min in the LEICA EM AC20 were analyzed in a JEOL TEM 200 keV JEM2100-Plus equipped with a 20-megapixel EMSIS Xarosa CMOS camera (EMSIS, Münster, Germany), or a Zeiss Leo Omega AB equipped with a CCD camera (Tröndle). To facilitate correlation, first low magnification overview images were recorded and compared to LM overview images. After identifying the ROI, high magnification images of corresponding cells were recorded.

### 4.11 TEM tomography acquisition

Prior to tomogram acquisition and contrasting, sections on grids were labeled with 10 or 15 nm protein-A gold (PAG) fiducials on both sides. For this, grids were incubated for 3 min on a 1:50 diluted drop of PAG with distilled water and subsequently washed thrice for 1 min with distilled water. Tilt series were acquired from +60° to −60° with 1° increments using the TEMography software (JEOL, Freising, Germany) on a JEM 2100-Plus system operating at 200 keV and equipped with a 20-megapixel CMOS camera. Tomograms were reconstructed using the back projection algorithm in IMOD [55].

### 4.12 Quantification of fluorescence

The fluorescence of intracellular HTL-conjugated dyes bound to Tom20-HaloTag was analyzed via the software FIJI, either directly after the first fixation of cells by means of sum projections of Z stacks of whole cells acquired over the same Z-range, or after EM sample preparation by means of single images of 250 nm EPON sections. The custom-made algorithm enhanced the contrast of the image, applied a Gaussian blur and subtracted the background before generating a mask by thresholding. Values measured of the area outside the selection were ascribed to the background. After manually deselecting areas with bright fluorescence, which obviously did not correspond to mitochondria but were rather induced by dust particles or folds in the section, the values of the remaining selection were measured. Lastly, cells were counted manually and the selections were saved as a separate image allowing subsequent clarification if necessary. If the selection clearly traced the mitochondria, the acquired data was further taken into account for analysis. If the algorithm randomly selected various patches over the entire image, the image was considered as not showing any fluorescence. Selections not selecting fluorescence signals within the cells or selecting many background speckles were excluded from further analysis.

### 4.13 Correlation of LM and TEM images

Correlation of single slices from the Z-stack obtained with LM were manually superimposed onto either single slices from the corresponding tomographic volume or single TEM images using Adobe Photoshop. In detail, TEM and LM images were opened within the same project and the opacity of the LM image was reduced. Consequently the LM image was transformed rididly or non-rigidly as also explained and utilized within the published software eC-CLEM [56].

## Data availability statement

Data will be made available on request.

## CRediT authorship contribution statement

**Rico Franzkoch:** Writing – original draft, Visualization, Validation, Resources, Methodology, Investigation, Formal analysis, Data curation. **Sabrina Wilkening:** Writing – original draft, Software, Methodology, Investigation, Formal analysis, Conceptualization. **Viktoria Liss:** Writing – original draft, Supervision, Investigation, Formal analysis, Data curation, Conceptualization. **Michael Holtmannspötter:** Resources, Methodology, Investigation, Formal analysis. **Rainer Kurre:** Resources, Methodology, Investigation, Formal analysis. **Olympia E. Psathaki:** Writing – original draft, Supervision, Project administration, Funding acquisition, Formal analysis, Conceptualization. **Michael Hensel:** Writing – review & editing, Visualization, Supervision, Resources, Project administration, Conceptualization.

## Declaration of competing interest

The authors declare that they have no known competing financial interests or personal relationships that could have appeared to influence the work reported in this paper.

The declaration of competing interests that you upload should be in a standard and editable format. Please select the suitable option and upload it with this submission. You can download the standard declaration of competing interests form from the following link.

https://declarations.elsevier.com/

Please note that all manuscripts submitted to Heliyon are checked for originality using the Crosscheck database (For more information on Crosscheck visit their website at http://www.crossref.org/crosscheck.html). We have studied your work carefully and have come to the conclusion that the textual overlap between your manuscript and previously published articles goes beyond the normal occurrence of standard phrases in your field. The largest overlap is with the following articles:

< https://doi.org/10.1101/2023.11.04.565612>

You can also find the largest sources of overlap in the attached iThenticate report. Please rewrite your manuscript to eliminate textual overlap with previously published literature before resubmitting. Please be aware that you will have one opportunity to address and reduce the overlap in your manuscript to an acceptable level. If you do not do this we may be forced to reconsider our decision for your submission and issue a reject decision.

Please reference all numbered tables in text. Currently, numbered table [4] in the manuscript have not been cited in text.

Notes to each check:

Plagiarism: Not Okay. 71% Overall; 65% < https://doi.org/10.1101/2023.11.04.565612>
