# Supplementary material for: Rapid in-EPON CLEM: Combining fast and efficient labeling of self-labeling enzyme tags with EM-resistant Janelia Fluor dyes and StayGold
Source: Heliyon. 2024 Mar 18;10(7):e28055. doi: 10.1016/j.heliyon.2024.e28055 (PMC10981041; doi:10.1016/j.heliyon.2024.e28055)
Supplement: Multimedia component 1 [file mmc1.pdf]

Rapid in-EPON CLEM: Combining fast and efficient labeling of self-labeling enzyme tags with EM-resistant Janelia Fluor dyes and StayGold

Rico Franzkoch [1,2], Sabrina Wilkening [1], Viktoria Liss [2], Michael Holtmannspötter [2,3], Rainer Kurre [2,3], Olympia E. Psathaki [2,3], Michael Hensel [1,3]

1 Abt. Mikrobiologie, Universität Osnabrück, Osnabrück, Germany, 2 iBiOs – Integrated Bioimaging Facility Osnabrück, 3 CellNanOs – Center for Cellular Nanoanalytics Osnabrück

Suppl. Figures and Figure Legends

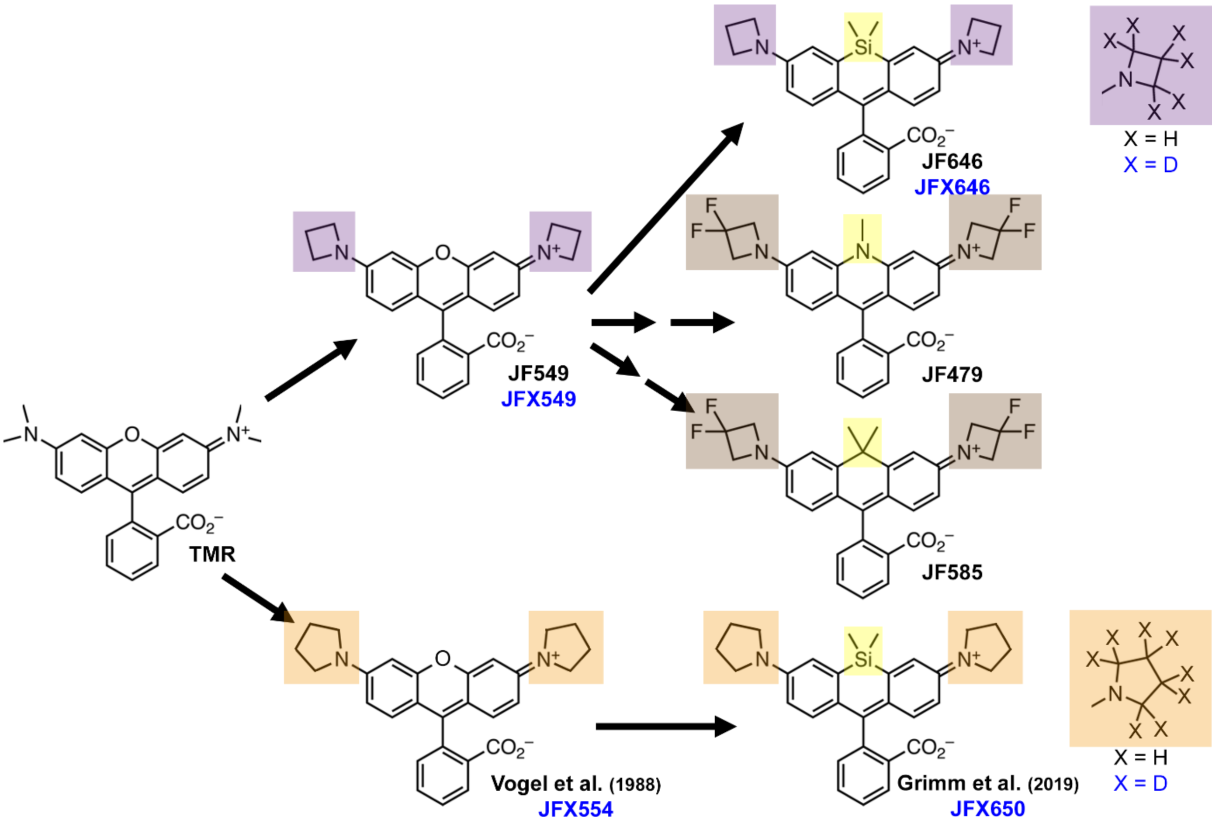

Fig. S 1. Origin of rhodamine-derived Janelia Fluor dyes. Substitution of the N-alkyl groups of tetramethylrhodamine (TMR) by azetidines (purple), pyrrolidine (orange) or a 3,3-difluoroazetidines (brown) together with replacing the oxygen atom of the xanthene by Si, N or C atoms (yellow) resulted in various dyes. Additional substitution of the hydrogen (H) by deuterium (D) in the azetidines and pyrrolidines improved the fluorescent characteristics of the dyes. The deuterated dyes are marked in blue, while the parent structures are marked in black. Structures shown were adapted from Janelia Materials (2023).

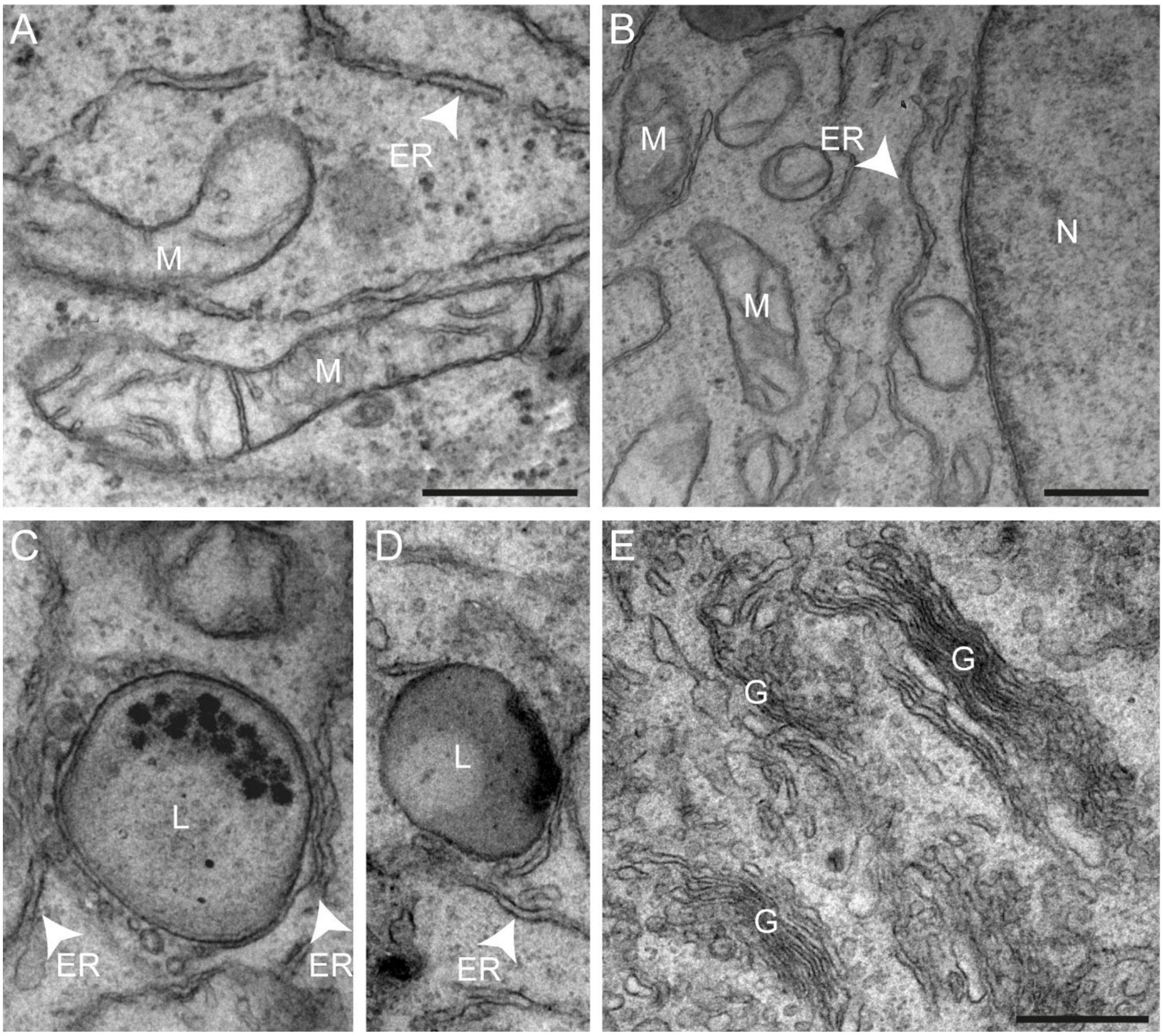

Fig. S 2. Ultrastructural preservation after OsO_4 treatment for 30 min. HeLa cells were conventionally prepared for EM as described in Material and Methods. Ultrathin sections were prepared to ensure good ultrastructural preservation even after the reduced duration of OsO_4 staining of 30 min. Several cellular structures including mitochondria (M) and ER (**A**), Nucleus (N) with double membrane (**B**), different stages of lysosomes and membrane contact sites (MCS) with ER (**C, D**), as well as parts of the Golgi apparatus (**E, G**) are well preserved. Arrowheads indicate potential MCS. Scale bars: A, B, E: 500 nm; C, D: 250 nm.

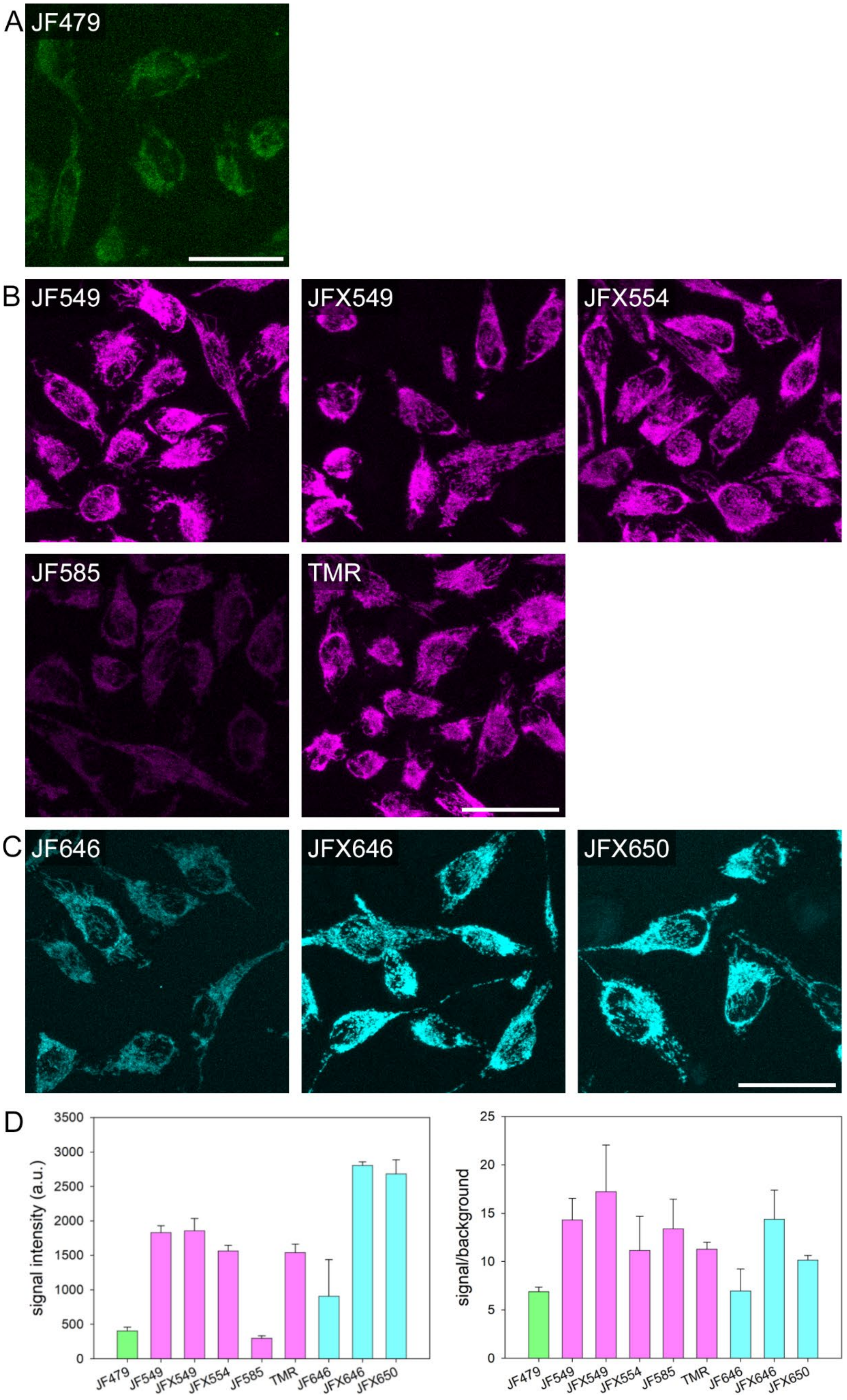

Fig. S 3. Specific labeling and sufficient fluorescence signal intensities were observed for most dyes tested. HeLa cells stably expressing Tom20-HaloTag were stained with 100 nM of

HTL conjugated to the indicated **A**) green, **B**) red, or **C**) far-red fluorescent dyes for 30 min. Fixation was performed with 3% PFA for 15 min. Image stacks of the same size were acquired using confocal laser-scanning microscopy (Leica SP5) with the same settings in the respective channel and were later processed the same way using maximum intensity projections. Scale bars: 50 μm. **D)** The average signal intensity was determined by the algorithm for selection and the background of each acquired image as described in Materials and Methods. Signal-to-background ratios (S/B) was computed by dividing the average signal intensity of the selection by the average signal intensity of the background for each image. Means and standard deviations of means values are shown,

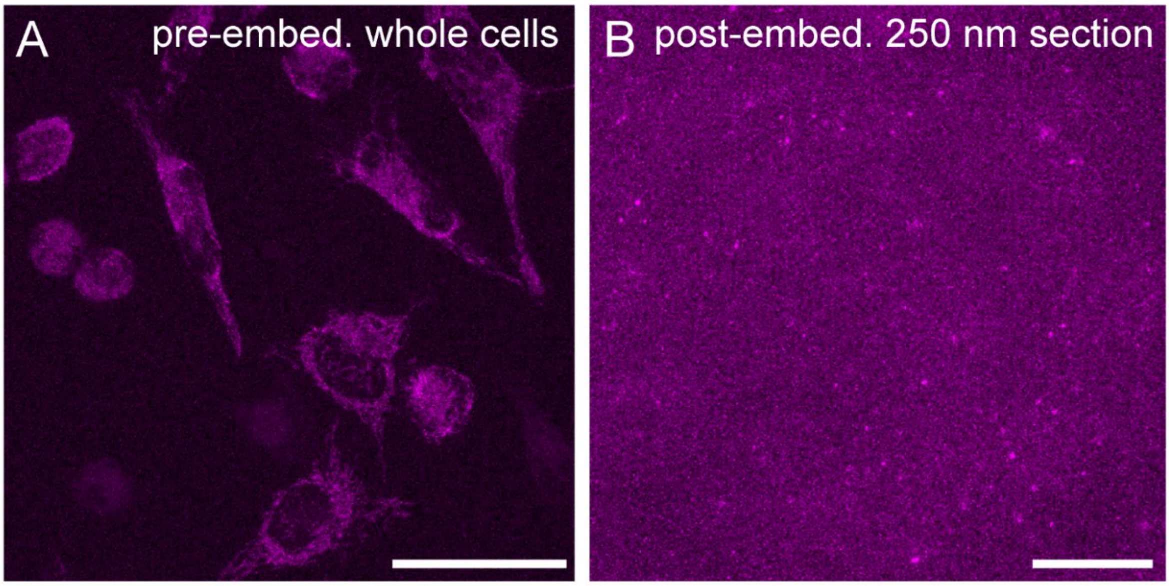

Fig. S 4. In-resin fluorescence is not retained at a higher JF585 concentration of 1 µM. A) Fluorescence image of living HeLa Tom20-HaloTag cells stained with 1 µM HTL-JF585 for 30 min. **B**) Fluorescence image of a 250 nm EPON section of HeLa Tom20-HaloTag cells stained with 1 µM HTL-JF585 for 30 min, fixed, conventionally prepared for EM and placed on a carbon-coated grid. Scale bars: A: 50 µm; B: 20 µm.

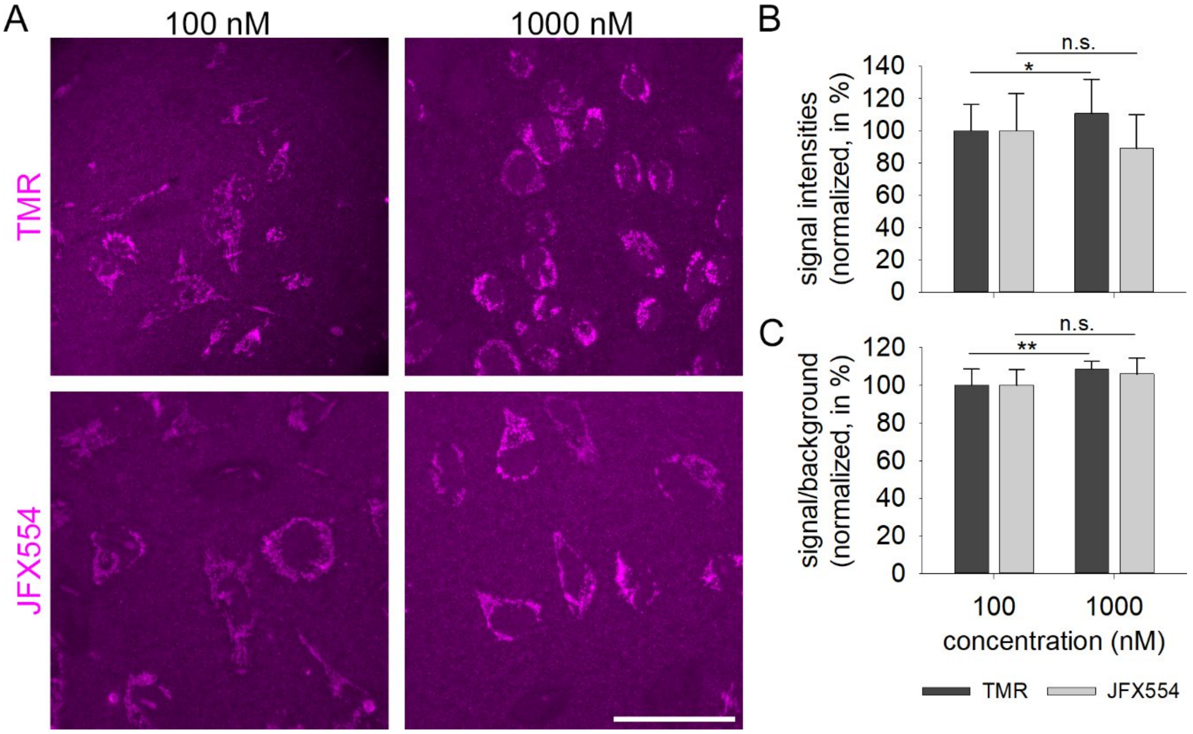

Fig. S 5. Increased dye concentrations improve in-resin fluorescence signals for TMR, but not for JFX554. HeLa Tom20-HaloTag cells stained with HTL-TMR or HTL-JFX554 in the indicated concentration for 30 min were conventionally prepared for EM as described for **Fig. 2**. **A**) Representative images of the respective dye and concentration. Scale bar, 50 µm. See **Movie 6** for Z series. **B**) Signal intensities and **C**) signal-to-background ratio (S/B) were obtained as described for **Fig. 3** and standardized by setting fluorescence signals obtained with 100 nM TMR or JFX554 to 100%. Statistical analyses were performed for each dye by unpaired, one-tailed t-test. Significances are indicated as follows: n.s., not significant, *, $p < 0.05$; **, $p < 0.01$; ***, $p < 0.001$.

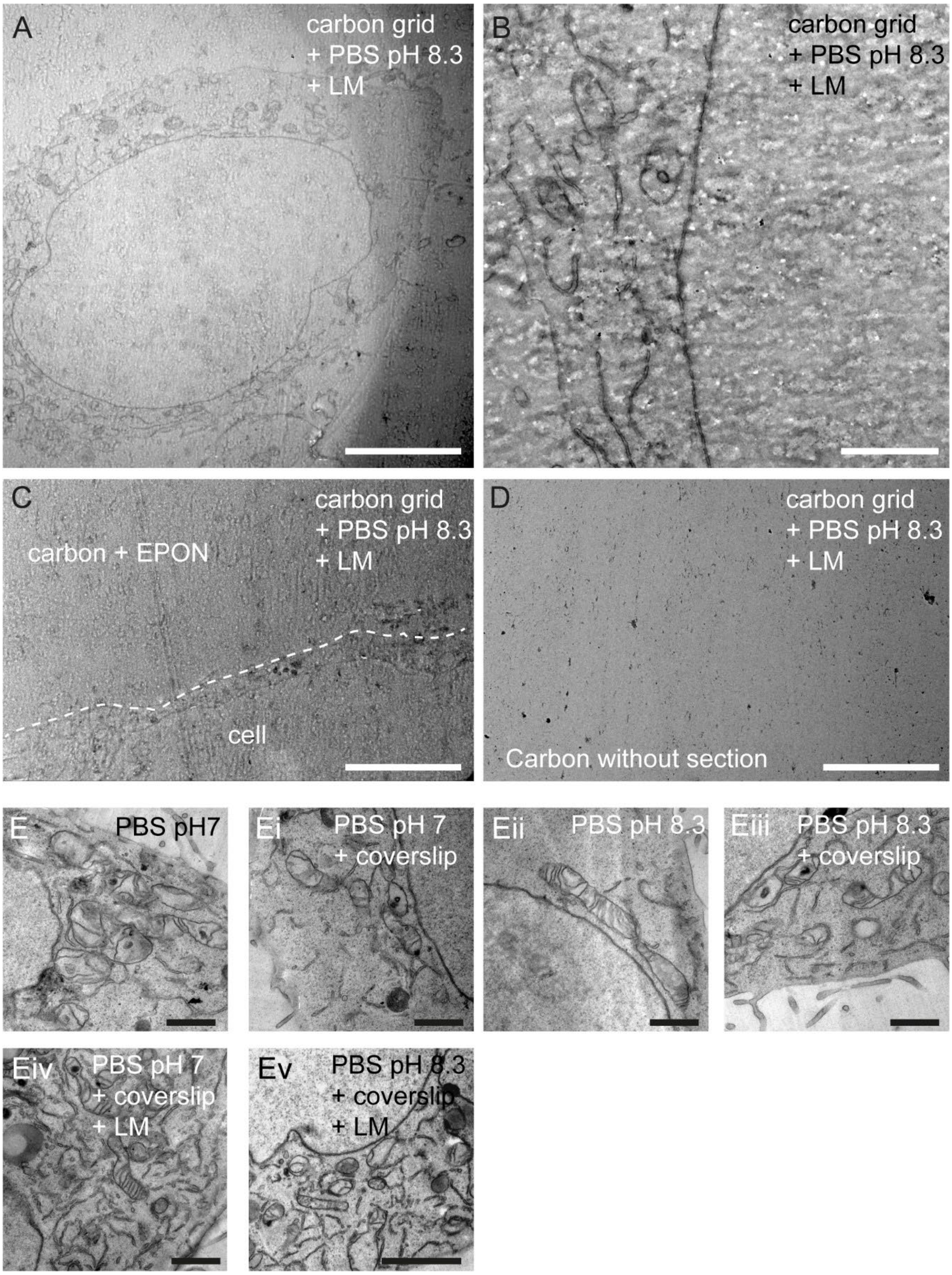

Fig. S 1. Artefacts induced after placing EPON sections on grids with carbon film only. HeLa cells were conventionally prepared for EM as described in Material and Methods. Ultrathin sections were prepared and mounted on commercial 200 mesh grids with carbon film only (**A–D**), or on custom-made Formvar coated grids (**E, Ev**). Grids were subjected to the standard LM imaging (**A–D, Ev**), but also the influence of a different pH of the buffer (**E, Eii**), the use of the coverslip sandwich mentioned in Material and Methods (**Ei, Eii**), as well as the LM itself were tested on deterioration of ultrastructure (**Eiv, Ev**). On grids with a carbon film,

the EPON shows severe artefacts indicates by wavy or holey shapes (**A, B**). These artifacts were not restricted to cellular material, but appeared also in empty EPON (**C**), but not on the carbon film without a section (**D**). Different pH of the buffer or imaging conditions did not influence the ultrastructure on formvar coated grids (**E– Ev**). This hints to a general problem of the combination of EPON and carbon-coated grids. Scale bars: A, C, D: 5 µm; B, E, Ei, Eii, Eiii, Eiv: 1 µm; Ev: 2 µm.

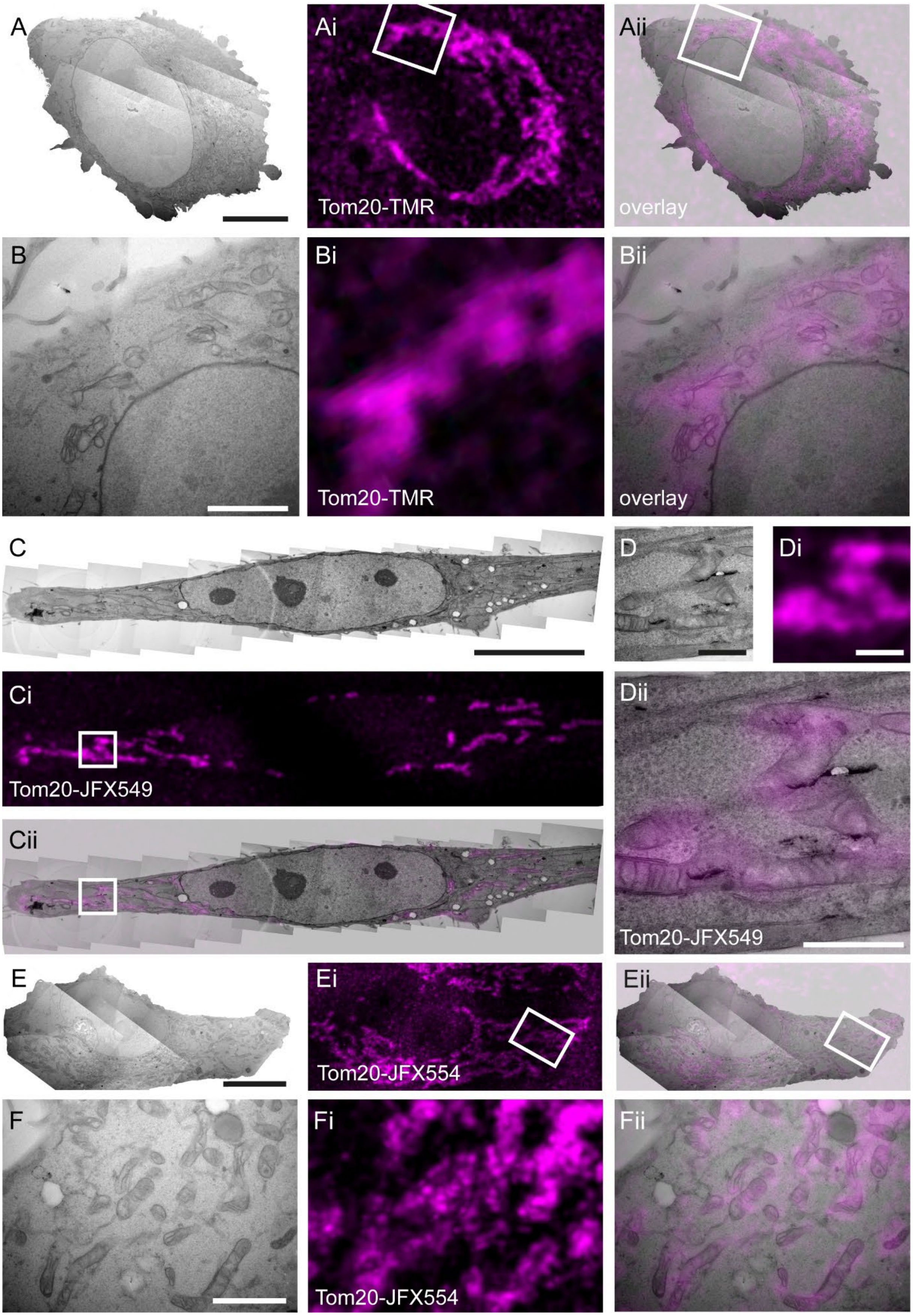

Fig. S 2. In-resin CLEM for Tom20-HaloTag with best-performing Janelia Fluor dyes and TMR using 250 nm sections. Hela Tom20-HaloTag cells were stained with 100 nM HTL-

TMR (**A, B**), HTL-JFX549 (**C, D**), or HTL-JFX554 (**E, F**) for 30 min. After conventional EM sample preparation, 250 nm semithin sections were prepared. Fluorescence signals were registered on sections using CLSM. All three dyes retained their fluorescence and allowed correlation to mitochondria identified in TEM modality. Scale bars: A, E: 10 µm; B, F: 2 µm; C: 5 µm; D, Dii: 500 nm.

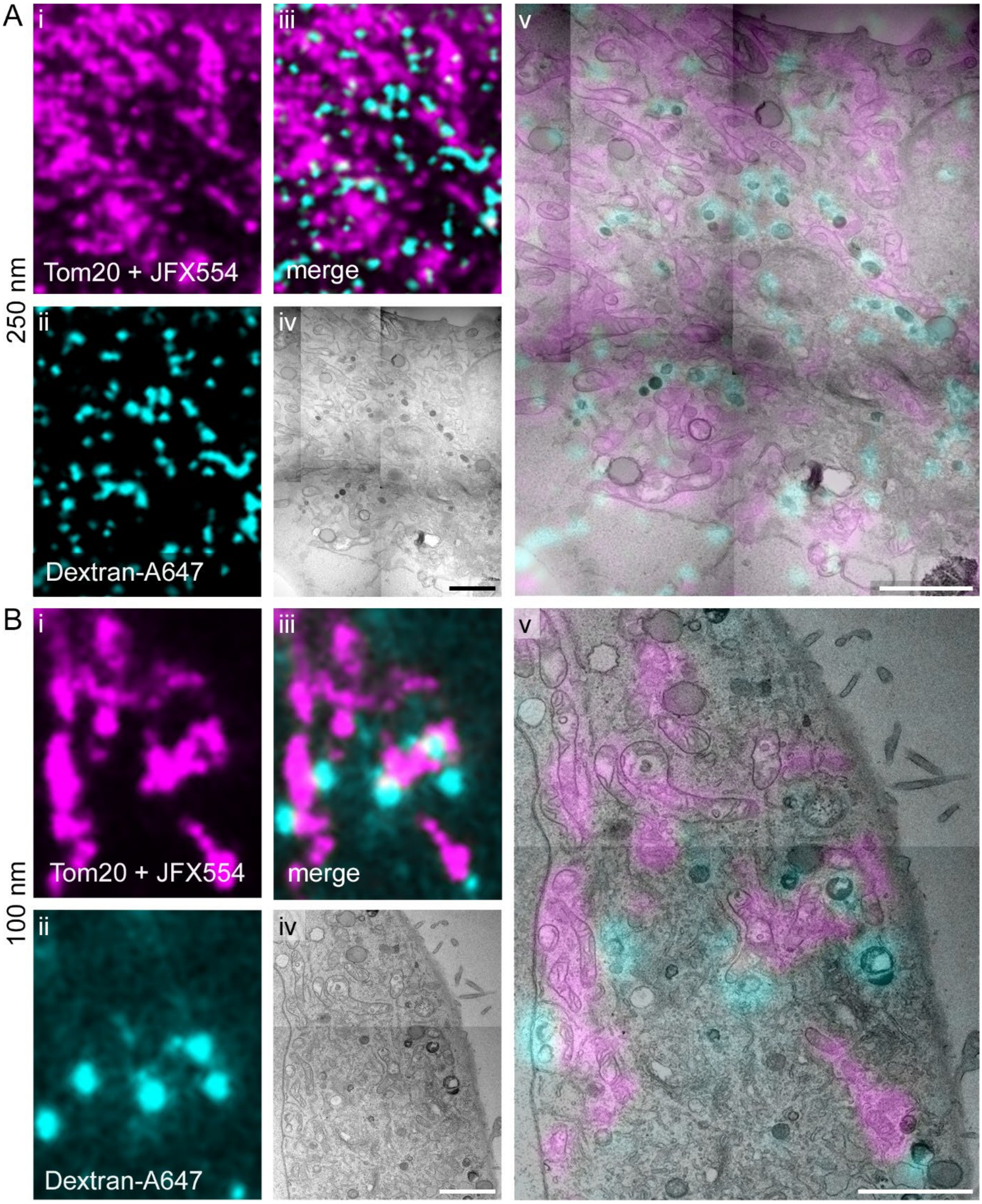

Fig. S 3. Dextran-Alexa Fluor 647 retains its fluorescence in EPON sections and is suitable for dual-fluorescence in-resin CLEM. Hela Tom20-HaloTag cells were pulsed with 100 µg/ml Dextran-Alexa Fluor 647 O/N and next day stained with 100 nM HTL-JFX554 for 30 min. Conventional EM sample preparation and imaging was conducted as described in Material and Methods. **A**) 250 nm section. **B**) 100 nm section. Dual-fluorescence CLEM and correlation of mitochondria and Alexa Fluor 647-containing endosomes was performed. Scale bars: 2 µm.

Supplemental Movies Captions

Movie 1: Tomogram corresponding to **Fig. 4B**

Movie 2: Tomogram corresponding to **Fig. 4F**

Movie 3: Tomogram corresponding to **Fig. 5E**

Movie 4: Tomogram corresponding to **Fig. 6**

Movie 5: Tomogram corresponding to **Fig. 7**

Movie 6: LM Z stack of EPON-embedded cells. The movie corresponds to data shown in **Fig. S 5A**

Supplemental Code

Source code of the algorithm used for quantitative comparison experiments

The source code below can be saved as a .ijm-file, implemented into Fiji under the menu bar Plugins>Macros>Install…, and used for measuring the fluorescence signal of mitochondria.

```
macro "Auswertung in-resin [q]" {

//get relevant image information
name=getTitle();
directory = File.directory;

//generate mask in a seperate window
run("Duplicate...", " ");
run("Enhance Contrast...", "saturated=0 normalize");
run("Gaussian Blur...", "sigma=3");
run("Subtract Background...", "rolling=50"); setAutoThreshold("IsoData dark");
setOption("BlackBackground", false);
run("Convert to Mask");
run("Create Selection");

//transfer selection to original window
selectWindow(name);
//setTool("rectangle");
run("Select None");
run("Restore Selection");

//measure background values
run("Make Inverse");
run("Set Measurements...", "area mean standard min integrated median display redirect=None
decimal=3");
run("Measure");
backarea=getResult("Area", nResults-1);
backmean=getResult("Mean", nResults-1);
backstddev=getResult("StdDev", nResults-1);
backmin=getResult("Min", nResults-1);
backmax=getResult("Max", nResults-1);
backintden=getResult("IntDen", nResults-1);
backmedian=getResult("Median", nResults-1);
backrawintden=getResult("RawIntDen", nResults-1);
run("Make Inverse");

//manually deselect areas of unwanted autofluorescence (created by dirt particles or by coverslip
background)
setTool("brush");
```

```
waitForUser("Brush", "Please remove dirt... \nTo adjust size of brush: double click on the brush
tool in the menu");
roiManager("add");

//measure fluorescence signal in selected area
run("Set Measurements...", "area mean standard min integrated median display redirect=None
decimal=3");
run("Measure");
setResult("background area",nResults-1,backarea);
setResult("background mean",nResults-1,backmean);
setResult("background StdDev",nResults-1,backstddev);
setResult("background Min",nResults-1,backmin);
setResult("background Max",nResults-1,backmax);
setResult("background IntDen",nResults-1,backintden);
setResult("background Median",nResults-1,backmedian);
setResult("background RawIntDen",nResults-1,backrawintden);
setResult("Label",nResults-1,name);
IJ.deleteRows(nResults-2, nResults-2);

//manually count cells
setTool("multipoint");
waitForUser("count cells", "Please mark all cells");
getSelectionCoordinates(xCoordinates, yCoordinates);
count=lengthOf(xCoordinates);
setResult("number of cells", nResults-1, count);

//save image with selection as .jpeg
roiManager("add");
roiManager("Select", 0);
roiManager("Set Color", "yellow");
roiManager("Select", 1);
roiManager("Set Color", "red");
roiManager("deselect");
roiManager("show all");
resetMinAndMax();
run("Capture Image");
print(directory);
print(name);
saveAs("Jpeg", directory+name+"_selection.jpeg");
close();
close(name);
close(substring(name,0,indexOf(name,".tif"))+"-1.tif");
roiManager("deselect");
roiManager("delete");
}
```
